# Weight‐reducing treatments are associated with an improvement in depression, functional health status, and quality of life: A meta‐analysis of randomized controlled trials

**DOI:** 10.1111/dom.70202

**Published:** 2025-10-23

**Authors:** Giovanni Antonio Silverii, Matteo Monami, Francesco Rotella, Maurizio De Luca, Tommaso Viozzi, Rocco Barazzoni, Silvio Buscemi, Luca Busetto, Paolo Sbraccia, Edoardo Mannucci

**Affiliations:** ^1^ AOU Careggi, Diabetology Unit – Experimental and Clinical Biomedical Sciences “Mario Serio” Department University of Florence Italy; ^2^ AOU Careggi, Psychiatry Unit, Department of Health Sciences University of Florence Italy; ^3^ Department of Medical, Surgical and Health Sciences University of Trieste Italy; ^4^ Department of Promozione della Salute, Materno‐Infantile, Medicina Interna e Specialistica di Eccellenza (PROMISE) University of Palermo Palermo Italy; ^5^ Department of Medicine University of Padova Padua Italy; ^6^ Department of Systems Medicine University of Rome Tor Vergata Rome Italy; ^7^ Department of Systems Medicine, Internal Medicine Unit – Obesity Center Teaching Hospital and University of Rome Tor Vergata Rome Italy

**Keywords:** antiobesity drug, bariatric surgery, eight management, meta‐analysis, obesity care, obesity therapy

## Abstract

**Aim:**

To assess whether there is a beneficial or detrimental effect of weight reduction on mental health.

**Materials and methods:**

Meta‐analysis of randomized trials performed for weight loss, in which weight loss at endpoint was greater than 5% in the intervention arm and smaller than 5% in the control arm, obtained with any surgical, endoscopic, or EMA‐approved pharmacological intervention. The endpoints were the incidence of overall and specific psychiatric adverse events.

**Results:**

Weight loss was associated with a reduced risk of major depression (MH‐OR 0.45 95% CI [0.21, 0.94], *I* ^2^ = 0), and overall depression (MH‐OR 0.72 [0.54, 0.97]); in subgroup analyses, a weight loss greater than 10% was associated with a lower incidence of depression than smaller weight loss (*p* = 0.04), whereas no difference was found between different interventions. No difference was detected in the incidence of anxiety (MH‐OR 1.04 [0.78, 1.39]), of serious (M‐H, OR CI 1.07 [0.78, 1.47]) and overall (MH‐OR 1.09 [0.89, 1.34]) psychiatric adverse events, suicidal ideation (M‐H, OR 0.87 [0.44, 1.70]), or suicide (M‐H, OR 0.87 [0.44, 1.70]). An improvement in functional health status was detected, either as SF‐36 Mental (SMD‐IV 0.45 [0.37, 0.52]) or SF‐36 Physical function (SMD‐IV 0.29 [0.14, 0.44]) or IWQOL Lite Physical function (MD‐IV 3.96 [1.60, 6.32]).

**Conclusion:**

Weight‐reducing treatments were associated with a beneficial effect on quality of life and functional health status and a reduced risk of depression, without any safety signal for serious or non‐serious psychiatric adverse events.

## 1 INTRODUCTION

Obesity is an increasingly common condition in which excess adiposity leads to several complications, including type 2 diabetes mellitus, sleep apnea, knee arthritis, and obesity‐associated cancers.^1^, ^2^, ^3^, ^4^ Obesity is also associated with a higher incidence of psychiatric disorders, particularly depression, with a bi‐directional relationship, that is, depression increasing the risk of obesity and vice versa.^5^ The pathophysiological mechanisms underlying this association are extremely complex and currently not fully understood.^6^ An altered reward system,^7^ inflammatory factors,^8^ and dysregulation of the hypothalamic–pituitary–adrenal axis^6^ are some examples of the possible factors involved. A weight loss of at least 3%–7% of baseline has been reported to provide substantial benefits in diabetes prevention and control of type 2 diabetes^7^ and other complications of obesity; hence, a 5% weight loss is widely accepted as the minimum threshold to acknowledge an obesity treatment as satisfactory.^8^ However, it is unclear whether weight loss may reduce the risk of depression and improve psychological and emotional well‐being. A growing number of surgical procedures and medications have been approved that are effective in providing weight loss; some of those, such as Bupropion‐Naltrexone, Phentermine‐Topiramate, glucagon GLP‐1RAs, and Tirzepatide, involve a direct drug effect on the central nervous system, and some concerns have been raised about the possible psychiatric adverse events of many currently used weight‐reducing drugs: there have been some reports of suicide in people receiving GLP‐1RAs,^9^ although no safety signals in this respect emerged from a meta‐analysis of randomized trials.^10^ Concerns about the long‐term psychiatric effects of Phentermine‐Topiramate have also been raised, leading, together with considerations of cardiovascular safety, to the refusal of approval by the European Medicines Agency (EMA).^11^ Moreover, it has been suggested that bariatric surgery may increase suicidality risk.^12^

All the abovementioned observations suggest that it is crucial to understand whether weight loss, achieved through various strategies, positively or negatively influences the psychological or psychiatric conditions of patients with obesity. The present meta‐analysis aimed, therefore, to assess whether, in patients with obesity, weight reduction obtained through approved treatments also improves psychological well‐being.

## 2 MATERIALS AND METHODS

This is a post hoc analysis of a meta‐analysis performed for the development of the European Association for the Study of Obesity (EASO) GRADE‐based guidelines on the pharmacological treatment of obesity. The meta‐analysis aimed to verify the efficacy and safety of EMA‐approved obesity management medications in outpatients affected by overweight or obesity (BMI > 27 kg/m^2^), in comparison with either placebo or lifestyle interventions, or no therapy. The full protocol of the meta‐analysis has been registered in the PROSPERO Database (CRD42024625338 identification code), with the principal endpoint being total body weight loss at the endpoint, and additional outcomes being the change in body weight and composition, weight‐related comorbidities, and serious adverse events.

### 2.1 Outcomes of interest

The outcomes of our post hoc analysis were: the difference in incident depression and anxiety between weight loss and control; the improvement in depression, anxiety, functional health status, and quality of life, as assessed through validated questionnaires; the incidence of serious and non‐serious psychiatric adverse events.

### 2.2 Inclusion criteria

Demonstration of a statistically significant, placebo‐corrected weight loss of at least 5% of baseline weight is a valid primary efficacy criterion that a weight‐reducing medication should reach to be approved by the EMA^13^ or the FDA.^14^ Therefore, to be included, a trial should report the difference in weight loss as a prespecified primary or secondary endpoint, and detect a weight loss at endpoint which must be greater than 5% in the intervention arm, and smaller than 5% in the control arm, thus only including those trials in which the intervention arm had reached a satisfactory weight reduction, according to most guidelines, and the control arm did not.^15^

Furthermore, the intervention assessed in the trial should be an EMA‐approved pharmacological treatment, an endoscopic bariatric procedure, or metabolic bariatric surgery for the primary analysis, and an EMA‐ or FDA‐approved pharmacological treatment, an endoscopic bariatric procedure, or metabolic bariatric surgery for the secondary analysis. The control group may consist of any placebo or medication, sham or actual procedure.

### 2.3 Study search

Studies were retrieved through a systematic search on PubMed and Embase, using “overweight”, “obesity”, and the names of the drugs or procedures approved for weight loss as keywords (see Table S1, in the supplementary materials, for the full search string), up to March 1st, 2025. Furthermore, any reference of potential interest retrieved by the investigators was added to the list of titles to be screened. Two investigators (G.A.S. and M.M.) independently screened the articles, whereas conflicts were resolved by a fifth investigator (E.M.).

### 2.4 Data extraction

The estimates for the variables of interest (incidence of any psychiatric adverse event at endpoint, means and standard deviations of any questionnaire scores, trial duration, age at baseline, Body Mass Index [BMI] at baseline, and percentage of females enrolled) were extracted into a predetermined electronic sheet. Data were retrieved from the principal publication, if available; otherwise, secondary publications and the clinicaltrials.gov registry were used to retrieve missing data. Four authors (G.A.S, M.M., F.R., T.V.) independently extracted data, whereas conflicts were resolved by a fifth investigator (E.M.).

### 2.5 Data analysis and quality assessment

Two of the investigators (G.A.S. and M.M.) assessed the risk of bias in RCTs using the revised Cochrane‐recommended tool,^16^ which includes five specific domains: (1) bias arising from the randomization process; (2) bias due to deviations from intended interventions; (3) bias due to missing outcome data; (4) bias in the measurement of the outcome; (5) bias in the selection of the reported result.

The results of these domains were graded as ‘low’ risk of bias, ‘high’ risk of bias, or ‘uncertain’ risk of bias. The aim was to assess the effect of assignment to intervention (the ‘intention‐to‐treat’ effect). For domains 3, 4, 5, the present meta‐analysis's primary endpoint, that is, the incidence of psychiatric serious and non‐serious adverse events, was considered as the outcome of interest. Any conflicts between the two investigators' evaluations were resolved by a third investigator (MM). We calculated the Mantel–Haenszel Odds Ratio [MH‐OR] for categorical variables, using random effect models in case of significant heterogeneity, and a fixed effect model if heterogeneity was not relevant. We performed a subgroup analysis for different weight loss medications or procedures: the prespecified subgroups were GLP‐1RAs (Semaglutide and Liraglutide), Tirzepatide, Bariatric Surgery or Endoscopy (encompassing all the surgical or endoscopic approved metabolic and bariatric procedures in Europe), Orlistat, and Naltrexone/Bupropion. When a significant difference between the weight loss and control groups was found, a subgroup analysis was performed for baseline BMI (a 40 kg/m^2^ threshold, will be used, to assess whether there is a difference in the effect of weight loss on mental health between mild to moderate obesity and severe obesity^17^), and the proportion of weight loss achieved at the end of the trial in the intervention arm.

## 3 RESULTS

### 3.1 Included studies

Out of 2646 items retrieved through the search of Medline and Embase, we selected 151 studies after reading titles and abstracts. However, 122 of these were excluded after reading the full text because the inclusion criteria were not met (see Figure S1, Table S2). We therefore identified 30 studies. The characteristics of the included trials are reported in Table 1. The weight reduction arm consisted of a pharmacological therapy in studies, of which five were with bariatric surgery (three with RYGB,^18^, ^19^, ^20^ one with ESG^21^ and DJBL,^22^ respectively), one on Orlistat,^23^ two with Phentermine/Topiramate,^24^, ^25^ three Naltrexone/Bupropion,^26^, ^27^, ^28^ five Tirzepatide,^29^, ^30^, ^31^, ^32^, ^33^ and 14 with GLP1‐RAs (six with Liraglutide^34^, ^35^, ^36^, ^37^, ^38^, ^39^ and eight with Semaglutide,^40^, ^41^, ^42^, ^43^, ^44^, ^45^, ^46^, ^47^ respectively). Definitions of psychiatric adverse events were heterogeneous across trials: all trials except two^21^, ^23^ used the Medical Dictionary for Regulatory Activities to define adverse events, with 7 and 10 studies also using questionnaire scores as regards to depression^21^, ^25^, ^27^, ^28^, ^29^, ^31^, ^33^, ^37^, ^45^, ^46^ and suicidality,^25^, ^29^, ^31^, ^33^, ^37^, ^45^, ^46^ respectively. In all trials except for three,^23^, ^26^, ^28^ psychiatric adverse events were systematically evaluated at each visit; furthermore, 13, 10, and 14 trials assessed depression,^21^, ^24^, ^25^, ^26^, ^27^, ^30^, ^31^, ^33^, ^37^, ^45^, ^46^, ^47^ suicidality^24^, ^25^, ^29^, ^30^, ^31^, ^33^, ^37^, ^45^, ^46^, ^47^ or quality of life,^19^, ^20^, ^27^, ^30^, ^33^, ^37^, ^38^, ^39^, ^40^, ^41^, ^43^, ^46^, ^47^ respectively, through specific questionnaires. The risk of bias of the study is reported in Figures S2 and S3; briefly, the risk of bias was low to moderate: 13 of the included studies had a mental health‐related prespecified outcome, whereas 17 may have some risk of bias due to missing outcome data or outcome measurement; furthermore, eight studies had issues with possible attrition.

**TABLE 1:** Characteristics of studies included in the meta‐analysis.

Study	Intervention	*N*	Control	*N*	Dur	BMI	Weight loss (%)	
							Interv	Contr
Abu Dayyeh^21^	ESG	77	LM	110	52	*30–40*	13.6	0.8
Allison^24^	Phentermine/Topiramate	751	Pbo	513	56	42.2	10.9	1.6
Apovian^26^	Naltrexone/Bupropion	992	Pbo	492	56	36.1	6.4	1.2
Aronne^29^	Tirzepatide	335	Pbo	335	52	30.5	5.5	0
Astrup^34^	Liraglutide	93	Pbo	98	52	34.8	7.5	1.7
Bliddal^40^	Semaglutide	271	Pbo	136	68	40.3	13.7	3.2
Caiazzo^22^	DJBL	49	LM	31	52	*>30*	9.7	2.1
Cohen^18^	RYGB	51	LM	49	104	*30–35*		
Davies^35^	Liraglutide	423	Pbo	212	56	37.2	6	2
Davies^41^	Semaglutide	807	Pbo	403	68	35.9	9.6	3.4
Gadde^25^	Phentermine/Topiramate	1492	Pbo	993	56	36.6	9.8	1.2
Garvey^36^	Liraglutide	195	Pbo	197	56	35.5	5.8	1.5
Garvey^42^	Semaglutide	152	Pbo	152	104	38.5	15.2	2.6
Garvey^30^	Tirzepatide	623	Pbo	315	72	36.1	13.8	3.3
Greenway^27^	Naltrexone/Bupropion	1142	Pbo	569	56	36.1	6.1	1.3
Hollander^28^	Naltrexone/Bupropion	333	Pbo	169	56	36.4	5.1	1.8
James^23^	Orlistat	23	Pbo	23	52	37.5	8.8	2.0
Jastreboff^31^	Tirzepatide	1896	Pbo	643	72	38	20	3.1
Kadowaki^43^	Semaglutide	299	Pbo	191	68	31.9	13.4	2.1
Leroux^37^	Liraglutide	1505	Pbo	749	156	38.9	6.1	1.9
Lincoff^44^	Semaglutide	8803	Pbo	8801	104	33.3	10	0.9
Loomba^32^	Tirzepatide	95	Pbo	48	52	35	8.8	0.2
Malhotra^33^	Tirzepatide	234	Pbo	235	52	39	18.7	2
Mingrone^19^	RYGB	20	LM	20	520	*>30*	28.8	4.7
O'Neill^38^	Liraglutide	103	Pbo	136	52	39.3	8.3	2.3
Rubino^45^	Semaglutide	253	Pbo	85	68	37.8	15.8	1.9
Schauer^20^	RYGB	50	LM	43	260	*27–43*	21.7	4.9
Wadden^39^	Liraglutide	212	Pbo	206	56	35.6	6.2	0.2
Wadden^46^	Semaglutide	1306	Pbo	655	68	38	17.6	4.9
Wilding^47^	Semaglutide	407	Pbo	204	68	37.9	14.9	2.4

Abbreviations: BMI, body mass index; Contr, control; DJBL, Duodenal‐Jejunal Bypass Liner; Dur, mean study duration (in weeks); ESG, endoscopic sleeve gastroplasty; Interv, intervention; LM, lifestyle modification; *N*, number of enrolled subjects; Pbo, placebo; RYGB, Roux‐en‐Y Gastric Bypass. When mean BMI at baseline is not reported, the reported range is displayed in Italics.

### 3.2 Psychiatric serious and non‐serious adverse events

Twenty‐seven studies, enrolling overall 38,932 patients, reported at least one psychiatric adverse event, either as a serious or non‐serious adverse event (AE); funnel plots ruled out publication bias for both outcomes (Figures S4 and S5); accordingly, Egger's regression did not detect a significant publication bias (Intercept −0.39, *p* = 0.34 for any AE; Intercept −0.16, *p* = 0.57 for serious AE). When considering only EMA‐approved medication, we did not observe any significant difference in Psychiatric AE between weight reduction treatments and controls (MH‐OR with 95% CI: 1.09 [0.89, 1.34]), with low heterogeneity (*I* ^2^ = 40%), without any significant difference across different medications or procedures (*p* = 0.36, Figure S6). These findings were confirmed when adding phentermine‐topiramate (Figure S7), with no difference between groups (MH‐OR 1.14 [0.94, 1.39]), a moderate heterogeneity (*I* ^2^ = 50%), but no difference across different medications or procedures (*p* = 0.45 between groups).

**FIGURE 1:**
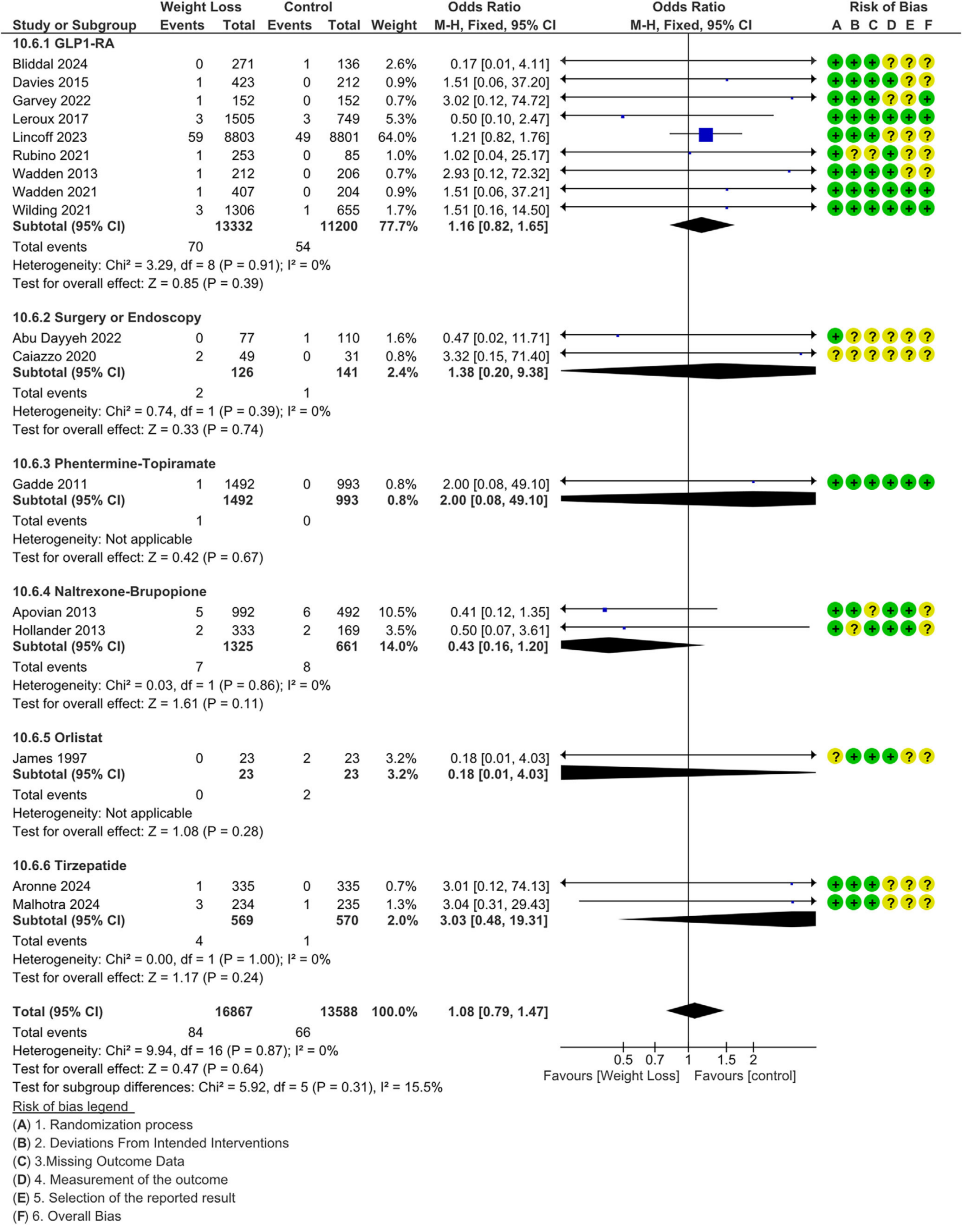
Difference in risk in psychiatric serious adverse events between weight loss and controls; FDA and EMA approved medications. M‐H, Mantel–Haenszel.

Sixteen studies reported at least one severe psychiatric adverse event (SAE). Weight loss was not associated with a difference in the risk for Psychiatric SAE (MH‐OR 1.07 [0.78, 1.47]), with low heterogeneity (*I* ^2^ = 40%), without any significant difference between different medications or procedures (*p* = 0.13, Figure S8). When phentermine/topiramate was also included, the figures remained similar (MH‐OR 95% CI 1.08 [0.79, 1.47]), with *p* = 0.31 for the difference between weight‐reducing treatments (Figure 1).

### 3.3 Depression

**FIGURE 2:**
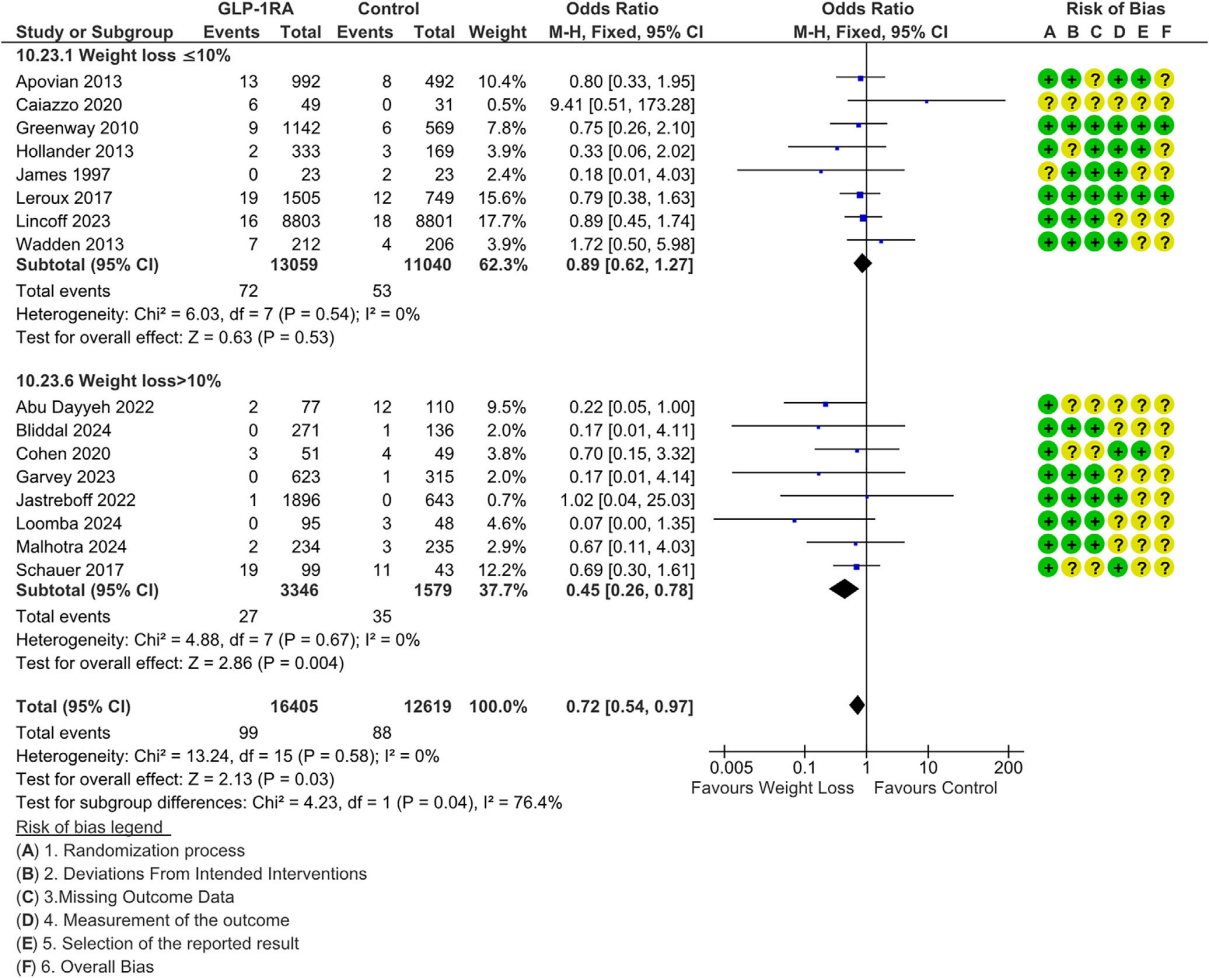
Difference in the incidence of depression between weight. M‐H, Mantel–Haenszel.

We retrieved 18 studies reporting the incidence of depression in weight loss and control arms; of those, four were performed on GLP‐1RAs, four on surgical or endoscopic procedures, three with Naltrexone/Bupropion, one with Orlistat, four with Tirzepatide, and two with Phentermine‐Topiramate. A funnel plot visual analysis ruled out the risk for publication bias (Figure S9), and Egger's regression confirmed the absence of significant publication bias (*p* = 0.15, intercept). When including only the EMA‐approved medications, weight‐reducing treatment was associated with a significant reduction in the incidence of depression (MH‐OR 0.72 [0.54, 0.97], *I* ^2^ = 0% Figure S10). Subgroup analyses did not detect any difference in effect when comparing different interventions (*p* = 0.49 for difference across groups, Figure S10), or when comparing patients with a baseline BMI lower or higher than 40 kg/m^2^ (*p* = 0.70 for difference between groups, Figure S11); on the other hand, a weight loss greater than 10% was associated with a significantly lower incidence of depression than a 5%–10% weight loss (*p* = 0.04 for difference between groups, Figure 2).

When including also Phentermine‐Topiramate, the effect of weight‐reducing treatment was no longer apparent (MH‐OR 0.99 [0.78, 1.25], Figure S12), with a significant difference between different interventions (*p* = 0.02, Figure S12). Phentermine‐topiramate was associated with a significant increase in the risk for depression in comparison with placebo (MH‐OR 1.65 [1.11, 2.46], Figure S12).

Twenty‐five cases of depression were reported as major depression in nine studies, none of which performed with phentermine‐topiramate: the risk of publication bias was not apparent in the funnel plot (Figure S13), and treatment for weight loss was associated with a reduced risk of major depression (MH‐OR 0.45, 95% CI [0.21, 0.94]), without any heterogeneity (*I* ^2^ = 0) or difference between different medications or techniques (*p* = 0.53, Figure S14).

Three studies, all performed with Bupropion/Naltrexone, reported Inventory Depression Scale (IDS) scores, showing a significant improvement in the active treatment arm (MD 0.83, 95% CI [0.46, 1.20], Figure S15).

### 3.4 Anxiety

Incident anxiety was reported in 17 studies, with no indication of publication bias emerging from a funnel plot (Figure S16), and the Egger's regression confirmed the absence of significant publication bias (*p* = 0.98, intercept 0.01). When only EMA‐approved medications were included, without any difference between weight loss treatment and comparators (MH‐OR 1.04, 95% CI [0.78, 1.39], *I* ^2^ = 0, *p* = 0.44 for the difference between interventions, Figure S17). When Phentermine‐Topiramate was included in the analysis (Figure S18), there was a trend toward an increase in the incidence of anxiety (MH‐OR 1.27, 95% CI [1.00, 1.62], *p* = 0.05), without significant heterogeneity between different interventions (*p* = 0.10). However, Phentermine‐Topiramate was the only intervention associated with increased anxiety (MH‐OR 1.92, 95% CI [1.23, 2.99], Figure S18).

### 3.5 Suicidal behaviour

Data on suicidal ideation was retrieved in nine studies, without any apparent publication bias (Figure S19), and the Egger's regression confirmed the absence of significant publication bias (*p* = 0.65, intercept −0.21); weight‐reducing treatment was not associated with any significant difference in risk for suicidal ideation in comparison with controls (MH‐OR 1.04, 95% CI [0.78, 1.39], Figure S20). Eight cases of committed suicide were detected in two trials, without any observed difference in risk between active treatment and comparators (MH‐OR 1.33, 95% CI [0.36, 4.91], Figure S21).

### 3.6 Functional health status and quality of life

**FIGURE 3:**
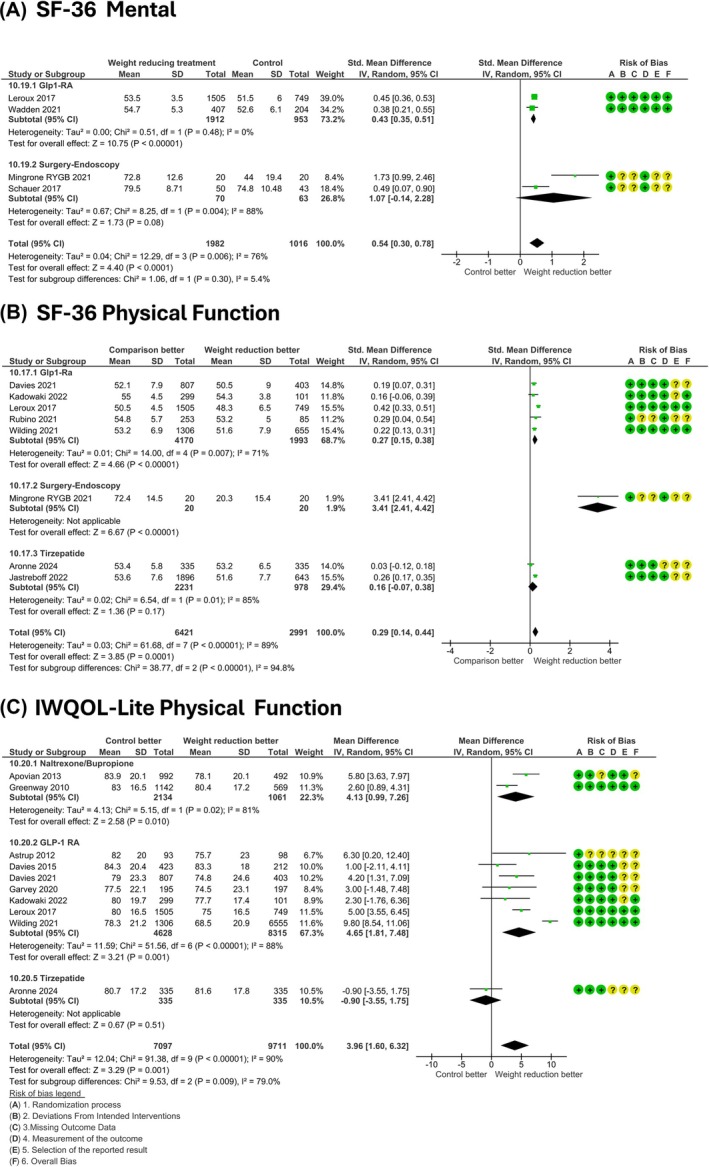
Difference in quality of life between weight‐reducing treatments and comparators; panel A and B: SF‐36, short form 36; panel C: IWQOL‐Lite, weight quality of life. IV, inverse variance; SD, standard deviation.

As for the Physical Function domain, nine studies assessed and reported data for Short Form‐36 Physical Function, without any suspected publication bias (Figure S20), and the Egger's regression confirmed the absence of significant publication bias (*p* = 0.36, intercept 2.43), detecting an improvement in the active treatment arm in respect to comparators (SMD‐IV, 95% CI 0.29 [0.14, 0.44]), Figure 3A. Whereas 10 studies assessed the Impact of Weight Quality Of Life Physical Function questionnaire, with observed higher scores in the intervention arm (MD‐IV, 95% CI 3.96 [1.60, 6.32], Figure 3B), with a possible risk for publication bias emerging from the funnel plot (Figure S21), which was ruled out by the Egger's regression (*p* = 0.52, intercept −3.44). Mental well‐being was assessed in four trials, enrolling 2998 patients, using the Short Form‐36 Mental domain; treatment for weight loss was associated with higher scores than comparators (SMD‐IV, 95% CI 0.45 [0.37, 0.52], Figure 3C).

### 3.7 Grading of evidence

The evidence was graded as moderate for major depression, anxiety, committed and ideated suicide, and low for functional health status and quality of life (Table S3).

## 4 DISCUSSION

The present meta‐analysis shows that treatments inducing weight loss are not associated with detrimental effects on mental health; conversely, when obtained with interventions free from proven negative psychiatric effects, weight loss is associated with a reduction in the incidence of depression, which is more evident when weight loss is greater than 10%. These observed results are consistent with a possible detrimental effect of excess weight on mental health. From a psychiatric standpoint, our results suggest an acceptable safety profile for most of the interventions considered, with no significant increase in psychiatric adverse events, including the risk of suicidal ideation or attempts. The only relevant exception is the combination of phentermine/topiramate, which is associated with increased rates of depression and anxiety. This particular profile, which emerges from a systematic assessment of available evidence from clinical trials, supports the decision of the EMA to deny approval for this treatment. In fact, although individual responses to different treatments could be variable across individuals, there is a relatively wide range of alternative drugs or procedures warranting similar or greater weight loss with a greater psychiatric safety.^48^

The improvement of depressive symptoms was greater in trials with a wider weight loss, whereas the psychological effects of weight‐reducing treatments appeared to be similar, irrespective of the treatments used. This finding suggests that weight loss per se could have beneficial effects on mood and psychological well‐being.

Overweight and obesity show a bi‐directional association with depression; beyond the neuroendocrine and inflammatory common mechanisms, psychological factors, such as the lack of self‐esteem and self‐efficacy, disturbed body image, and social stigma, may also play a role in this association, and all these factors could be ameliorated by weight loss. Notably, although many of the factors cited above could also increase levels of anxiety, weight loss does not seem to have relevant effects on anxiety. Physical function improvement, which in our meta‐analysis is significantly greater in those on weight‐reducing treatments, may contribute to the amelioration of mood, both directly and through the enhancement of physical activity. On the other hand, some potential detrimental effects of weight‐reducing treatments on mood seemed to be ruled out. First of all, side effects of drugs and surgical or endoscopic interventions do not seem relevant in modifying mood and quality of life. Furthermore, the reduction in food intake, which is the main mechanism through which the treatments included produce a weight reduction, may theoretically worsen patients' mood by impairing eating as a source of pleasure and reward, but the results of the present meta‐analysis suggest that, in subjects with obesity enrolled in trials aimed at weight reduction, the reduction of pleasure associated with eating may not play a decisive role.

Some weight‐reducing medications (i.e., orlistat) are devoid of direct central effects, implicating that any observed psychological effect is determined by weight loss. However, the paucity of data on this molecule prevents any definitive conclusions. On the other hand, other agents could theoretically modify the psychological status of patients: bupropion has anti‐depressant effects, which could be partly counterbalanced by naltrexone, whereas phentermine could increase anxiety. The absence of heterogeneity in the psychological effects across different weight‐reducing treatments (excluding phentermine/topiramate) may also rule out a direct neurotrophic effect of GLP‐1 RAs or Tirzepatide on the central nervous system as a determinant of its beneficial effect on mood, thus suggesting that the observed mood improvement in those receiving these medications may merely depend on BMI reduction. On the other hand, weight loss after metabolic surgery is also often associated with an increase in GLP‐1 levels^49^; therefore, a possible beneficial direct neurotrophic effect of GLP‐1 RAs may still be present, which would be plausible due to GLP‐1R observed effect on neurogenesis in animal models,^50^ and its possible interplay with dopamine, glutamate, and γ‐Aminobutyric acid neurotransmitter systems in the brain,^51^ which may in turn affect mood improvement. Furthermore, it has been recently observed that antidepressant medications, such as bupropion, may act through Brain‐Derived Neurotrophic Factor release,^52^ which is also stimulated by weight loss,^53^ and GLP‐1.^54^

Our meta‐analysis has some limitations, the most relevant being the heterogeneity in reporting psychiatric outcomes across different trials; some studies actively screened patients for suicidality risk, depression, anxiety, or modifications in quality of life, whereas in most cases, psychiatric adverse events and outcomes were registered only in case they emerged during clinical assessments. This necessitates, firstly, a more systematic assessment of the psychopathological status of patients enrolled in weight‐loss trials; and secondly, the development of a psychiatric assessment method that is both accurate and compatible with internal medicine and non‐psychiatric settings. Furthermore, included trials tend to exclude patients with major pre‐existing psychiatric disorders, which are more often associated with weight gain, due to the common detrimental lifestyle and the long‐term metabolic effects of psychotropic drugs. Further trials enrolling also patients with psychiatric conditions are needed to establish the safety of treatments for obesity in this specific population, often requiring weight‐reducing interventions. The effects of obesity treatments in this specific, but significant portion of the general population are still not fully addressed. In addition, the short follow‐up duration limits the evaluation of the long‐term impact on psychological status, especially in the case of weight regain. The assessment of the possible effect of weight regain on mental health is challenging because mental health diseases such as anxiety or depression may either be a predisposing factor of weight regain, due to a reduced adherence to prescribed diet, or its consequence,^55^ because of the possible detrimental effect on self‐esteem of failure of dietary intervention. The discrimination of the direction of the effect may not be acknowledged through observational studies, and it would likely require clinical trials designed for this purpose.

To better understand the complex interconnection between obesity and psychopathology, future prospective studies should include a more accurate design with a systematic assessment of mental health and the evaluation of the neurobiological mechanisms underlying the relationship between weight loss and mental well‐being.

## AUTHOR CONTRIBUTIONS

G.A.S., M.M., and E.M. made the analysis plan, researched data, performed analyses, contributed to the discussion, and wrote the first draft of the manuscript. F.R., T.V., M.D.L., R.B., S.B., L.B., and P.S. contributed to data research and reviewed and edited the manuscript. All the authors had full access to study data, approved the final version of the manuscript, and took responsibility for data integrity and analysis accuracy.

## FUNDING INFORMATION

This research was performed as part of the institutional activity of the unit, with no specific funding.

## CONFLICT OF INTEREST STATEMENT

G.A.S. has received a fee from Eli‐Lilly as a member of the advisory board; M.M. has received speaking fees from Astra Zeneca, Boehringer‐Ingelheim, Eli‐Lilly, Merck, Novo Nordisk, and Sanofi. S.B. has received consultancy or speaking fees or research contributions from Novo Nordisk, Eli Lilly, Boehringer, Pfizer, Dompè, Bruno Pharmaceuticals, Recordati Rare Diseases, Therascience, Merieux; L.B. has received consultancy fees from Novo Nordisk, Eli Lilly, Boehringer Ingelheim, Pfizer, Roche, Regeneron, and Bruno Farmaceutici and speaking fees from Rhythms Pharmaceuticals and Pronokal. E.M. has received consultancy fees or speaking fees or research contributions from AstraZeneca, Bayer, Boehringer‐Ingelheim, Coresearch, Dexcom, Eli‐Lilly, Molteni, Novo Nordisk, Pidkare, and Sanofi. P.S. received payment of honoraria and consulting fees from Boehringer Ingelheim, Chiesi, Novo Nordisk, Eli Lilly, Pfizer, and Roche as a member of advisory boards.

M.D.L., R.B., T.V., and F.R. do not have any competing interests to disclose.

## Supporting information


**Appendix S1:** Supplementary data.

## Data Availability

Data sharing not applicable to this article as no datasets were generated or analysed during the current study.
