# Supplementary material for: Weight‐reducing treatments are associated with an improvement in depression, functional health status, and quality of life: A meta‐analysis of randomized controlled trials
Source: Diabetes Obes Metab. 2025 Oct 23;28(1):347–57. doi: 10.1111/dom.70202 (PMC12673451; doi:10.1111/dom.70202)
Supplement: Supplementary file 1 — Appendix S1: Supplementary data. [file DOM-28-347-s001.pdf]

Weight-reducing treatments are associated with an improvement in depression and quality of life: a meta-analysis of randomized controlled trials.

**PUBMED ("obeses"[All Fields] OR "obesity"[MeSH Terms] OR "obesity"[All Fields] OR "obese"[All Fields] OR "obesities"[All Fields] OR "obesity s"[All Fields] OR ("overweight"[MeSH Terms] OR "overweight"[All Fields] OR "overweighted"[All Fields] OR "overweightness"[All Fields] OR "overweights"[All Fields])) AND ("orlistat"[Supplementary Concept] OR "orlistat"[All Fields] OR "orlistat"[MeSH Terms] OR "orlistat s"[All Fields] OR ("naltrexone"[Supplementary Concept] OR "naltrexone"[All Fields] OR "naltrexon"[All Fields] OR "naltrexone"[MeSH Terms] OR "naltrexone s"[All Fields]) OR ("bupropion"[Supplementary Concept] OR "bupropion"[All Fields] OR "amfebutamone"[All Fields] OR "bupropion"[MeSH Terms] OR "bupropion s"[All Fields] OR "bupropione"[All Fields]) OR "topiramate phentermine"[All Fields] OR ("liraglutid"[All Fields] OR "liraglutide"[Supplementary Concept] OR "liraglutide"[All Fields] OR "liraglutide"[MeSH Terms] OR "liraglutide s"[All Fields]) OR ("semaglutide"[Supplementary Concept] OR "semaglutide"[All Fields]) OR ("tirzepatide"[Supplementary Concept] OR "tirzepatide"[All Fields] OR "tirzepatide"[MeSH Terms])) OR "obesity surgery"[All Fields] OR "bariatric surgery"[MeSH Terms] OR ("bariatric"[All Fields] AND "surgery"[All Fields]) OR "bariatric surgery"[All Fields] OR "Sleeve Gastrectomy or Roux en Y" [All Fields] OR "Gastric Bypass" [All Fields] or "One Anastomosis Gastric Bypass"[All Fields] or "Laparoscopic Adjustable Gastric Banding" [All Fields] or "Bilio-Pancreatic Diversion" [All Fields] or "Single Anastomosis Duodenal–Ileal bypass" [All Fields] or "Intragastric Balloons or Primary Obesity Surgery Endoluminal" [All Fields] or Endoscopic Sleeve Gastroplasty or aspiration therapy or Duodenal–Jejunal Bypass Liner (DJBL) or lifestyle interventions)"**	
**Embase** (**'obesity'**/exp OR **obesity** OR **'overweight'**/exp OR **overweight**) AND (**'orlistat'**/exp OR **orlistat** OR **'naltrexone bupropion'**/exp OR **'naltrexone bupropion'** OR ((**'naltrexone'**/exp OR **naltrexone**) AND (**'bupropion'**/exp OR **bupropion**)) OR **'topiramate fentermine'** OR ((**'topiramate'**/exp OR **topiramate**) AND **fentermine**) OR **'liraglutide'**/exp OR **liraglutide** OR **'semaglutide'**/exp OR **semaglutide** OR **'tirzepatide'**/exp OR **tirzepatide**) ***('surgery'/exp OR surgery) AND [embase]/lim NOT ([embase]/lim AND [medline]/lim) "bariatric surgery"[MeSH Terms] OR ("bariatric"[All Fields] AND "surgery"[All Fields]) OR "bariatric surgery"[All Fields]***	

Table S1 – Information on search string

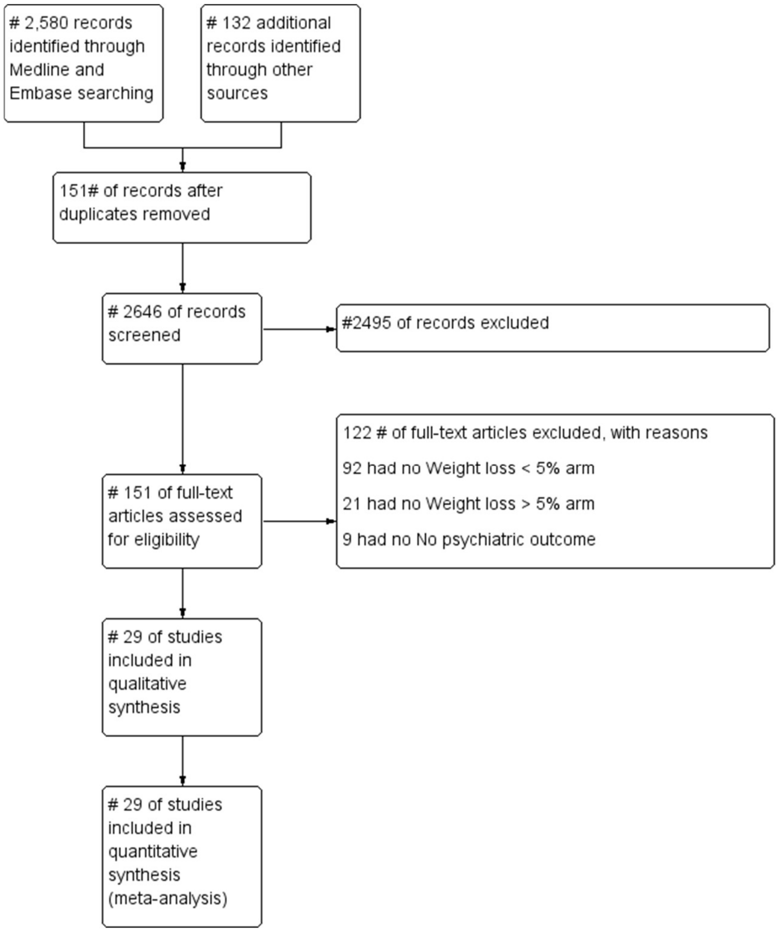

Figure S1: trial flow summary.

	1. Randomization process	2. Deviations From Intended Interventions	3.Missing Outcome Data	4. Measurement of the outcome	5. Selection of the reported result	6. Overall Bias
Abu Dayyeh 2022	+	?	?	?	?	?
Allison 2012	+	+	?	+	+	?
Apovian 2013	+	+	?	+	+	?
Aronne 2024	+	+	+	?	?	?
Astrup 2012	+	?	?	?	?	?
Bliddal 2024	+	+	+	?	?	?
Caiazzo 2020	?	?	?	?	?	?
Cohen 2020	+	?	?	+	+	?
Davies 2015	+	+	+	+	?	?
Davies 2021	+	+	+	+	?	?
Gadde 2011	+	+	+	+	+	+
Garvey 2020	+	+	+	+	?	?
Garvey 2022	+	+	+	?	?	+
Garvey 2023	+	+	+	?	?	?
Greenway 2010	+	+	+	+	+	+
Hollander 2013	+	?	+	+	+	?
James 1997	?	+	+	+	?	?
Jastreboff 2022	+	+	+	+	?	?
Kadowaki 2022	+	+	+	+	?	+
Leroux 2017	+	+	+	+	+	+
Lincoff 2023	+	+	+	?	?	?
Loomba 2024	+	+	+	?	?	?
Malhotra 2024	+	+	+	?	?	?
Mingrone 2021	+	?	?	+	?	?
O' Neill 2018	+	+	+	+	?	+
Rubino 2021	+	?	?	+	?	?
Schauer 2017	+	?	?	+	?	?
Wadden 2013	+	+	+	+	?	?
Wadden 2021	+	+	+	+	+	+
Wilding 2021	+	+	+	+	+	+

Figure S2: *Risk of bias summary*

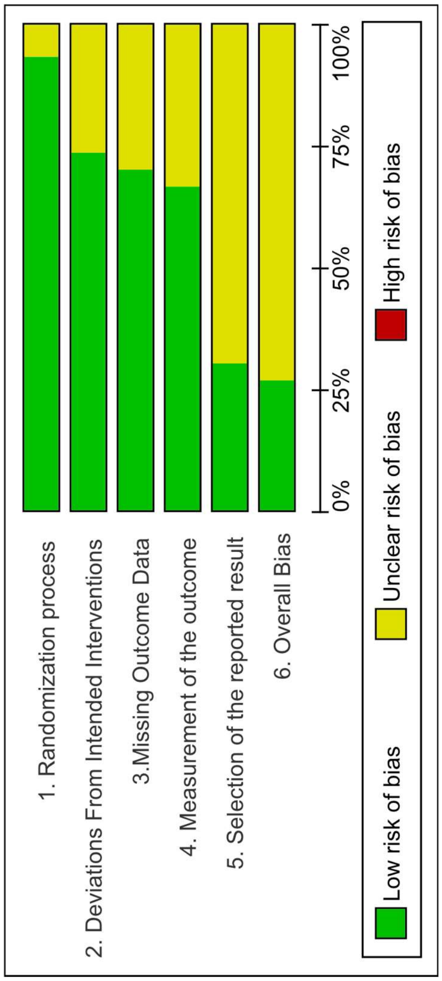

Figure S3: *Risk of bias graph*

Table S2: list of excluded trials, with reasons for exclusion.

#	Trial name	Reason for exclusion	#	Trial name	Reason for exclusion
1	Derosa 2012	No Weight loss > 5% arm	62	Davies 2021	No outcome
2	Gudbergsen 2021	No Weight loss > 5% arm	63	Derosa 2012	No outcome
3	Hauptman 2000	No Weight loss > 5% arm	64	Dixon 2008	No outcome
4	Karhunen 2000	No Weight loss > 5% arm	65	Dowsey 2022	No outcome
5	Kelley 2002	No Weight loss > 5% arm	66	Feigel-Guiller 2015	No outcome
6	Miles 2002	No Weight loss > 5% arm	67	Garvey 2020	No outcome
7	Nissen 2016	No Weight loss > 5% arm	68	Hollander 1998	No outcome
8	Richelsen 2007	No Weight loss > 5% arm	69	Kadowaki 2022	No outcome
9	Sullivan 2017	No Weight loss > 5% arm	70	Karhunen 2000	No outcome
10	Davidson 1999	No Weight loss < 5% arm	71	Kosiborod 2023	No outcome
11	Finer 2000	No Weight loss < 5% arm	72	Kosiborod 2024	No outcome
12	Rossner 2000	No Weight loss < 5% arm	73	Krempf 2003	No outcome
13	Mingrone 2021	No Weight loss < 5% arm	74	Liang 2013	No outcome
14	Salte 2024	No Weight loss < 5% arm	75	McGowan 2024	No outcome
15	Schauer ter 2017	No Weight loss < 5% arm	76	Mingrone 2021b	No outcome
16	Ruban 2022	No Weight loss < 5% arm	77	Mingrone 2021c	No outcome
17	Schauer 2017	No Weight loss < 5% arm	78	Astrup (a) 2012	No outcome
18	Schauer bis 2017	No Weight loss < 5% arm	79	Bakris 2002	No outcome
19	Coffin 2017	No Weight loss < 5% arm	80	Berne 2005	No outcome
20	Wadden 2021	No Weight loss < 5% arm	81	Bliddal 2024	No outcome
21	Davidson 1999	No Weight loss < 5% arm	82	Cheng 2020	No outcome
22	*Morino* 2003	No Weight loss < 5% arm	83	Roushdy 2020	No Weight loss < 5% arm
23	*Lundell* 1997	No Weight loss < 5% arm	84	Hany 2024	No Weight loss < 5% arm
24	*Nilsell* 2001	No Weight loss < 5% arm	85	Axer 2024	No Weight loss < 5% arm
25	*Scozzari* 2009	No Weight loss < 5% arm	86	Talebpour 2018	No Weight loss < 5% arm
26	*Jain* 2021	No Weight loss < 5% arm	87	Robert 2024	No Weight loss < 5% arm
27	*Musella* 2021	No Weight loss < 5% arm	88	Karagul 2024	No Weight loss < 5% arm
28	*Kehagias*2011	No Weight loss < 5% arm	89	Catheline 2019	No Weight loss < 5% arm
29	*Level* 2021	No Weight loss < 5% arm	90	Nguyen 2018	No Weight loss < 5% arm
30	*Biter* 2017	No Weight loss < 5% arm	91	Darabi 2013	No Weight loss < 5% arm
31	*Eskandaros* 2021	No Weight loss < 5% arm	92	Delko 2024 1	No Weight loss < 5% arm
32	*Ignat* 2017	No Weight loss < 5% arm	93	Karamanakos 2008	No Weight loss < 5% arm
33	*Lee* 2004	No Weight loss < 5% arm	94	Verrastro 2023	No Weight loss < 5% arm
34	*MacLean* 1993	No Weight loss < 5% arm	95	Yang 2015	No Weight loss < 5% arm
35	*Ramon* 2012	No Weight loss < 5% arm	96	O'Brien 20131	No Weight loss < 5% arm
36	*Skroubis* 2014	No Weight loss < 5% arm	97	Paluszkiewicz 2012	No Weight loss < 5% arm
37	*Olbers* 2005	No Weight loss < 5% arm	98	Pajecki 2023	No Weight loss < 5% arm
38	*Grubnik* 2016	No Weight loss < 5% arm	99	Svanevik 2023	No Weight loss < 5% arm
39	Zhang 2014	No Weight loss < 5% arm	100	O'Neil 2018	No Weight loss < 5% arm
40	Hall bis 1990	No Weight loss < 5% arm	101	Packer 2024	No Weight loss < 5% arm
41	Hall 1990	No Weight loss < 5% arm	102	Peterli 2018	No Weight loss < 5% arm
42	Hall ter 1990	No Weight loss < 5% arm	103	Dixon a2012	No Weight loss < 5% arm
43	Casajoana 2021	No Weight loss < 5% arm	104	Courcolas ter 2020	No Weight loss < 5% arm
44	Casajoana c2021	No Weight loss < 5% arm	105	Lee 2014	No Weight loss < 5% arm
45	Wallenius2020	No Weight loss < 5% arm	106	Ikramuddin 2018	No Weight loss < 5% arm
46	Salminen2022	No Weight loss < 5% arm	107	Petry 2015	No Weight loss < 5% arm
47	Singh2023	No Weight loss < 5% arm	108	Salte 2024	No Weight loss < 5% arm
48	Poston 2003	No Weight loss < 5% arm	109	Casajoana bis 2021	No Weight loss < 5% arm
49	Tang 2016	No Weight loss < 5% arm	110	Hedberg 2012	No Weight loss < 5% arm
50	Sullivan 2013	No Weight loss < 5% arm	111	Ruban 2022	No Weight loss < 5% arm
51	Richelsen 2007	No Weight loss < 5% arm	112	Abu Dayyeh 2021	No Weight loss < 5% arm
52	Schauer ter 2017	No Weight loss < 5% arm	113	Fuller 2013	No Weight loss < 5% arm
53	Rubino 2021	No Weight loss < 5% arm	114	Miller 2017	No Weight loss < 5% arm
54	*Courcolas b*2020	No Weight loss < 5% arm	115	*Spaggiari* 2021	No Weight loss < 5% arm
55	*Schauer* 2017	No Weight loss < 5% arm	116	*Thompson* 2017	No Weight loss < 5% arm
56	*Glaysher* 2017	No Weight loss < 5% arm	117	*Dargent* 2015	No Weight loss < 5% arm
57	*Schauer bis* 2017	No Weight loss < 5% arm	118	*Loomba* 2023	No Weight loss < 5% arm
58	*Schiavon* 2020	No Weight loss < 5% arm	119	*Ponce (a)* 2015	No Weight loss < 5% arm
59	*Courcoulas* 2017	No Weight loss < 5% arm	120	_Sullivan**_ 2018	No Weight loss < 5% arm
60	*Simonson 2019*	No Weight loss < 5% arm	121	*Koehestanie 2014*	No Weight loss < 5% arm
61	*Zhang 2014*	No Weight loss < 5% arm	122	*Xiang 2018*	No Weight loss < 5% arm

Study	Psychiatric AE Definition	Psychiatric AE Ascertainment Method
Abu Dayyeh 2022[1]	Depression: PHQ-9> 10 Other: investigator judgemen	Depression: systematic PHQ-9 Other: AE Systematic evaluation
Allison 2012[2]	AE: MedDRA 10.1	Depression, suicidality: Systematic PHQ-9, C-SSRS. Other: AE systematic evaluation
Apovian 2013[3]	Medra 15.0	Depression: systematic IDS-SR<br>Other: reported by patient
Aronne 2024[4]	AE: MedDRA 26.0 Depression: PHQ-9> 15 Suicidality C-SSRS	Depression, suicidality: Systematic PHQ-9, C-SSRS Other: AE systematic evaluation
Astrup 2012[5]	MedDRA coding 10.1	AE systematic evaluation
Bliddal 2024 [6]	MedDRA coding 26	SF-36; AE systematic evaluation
Caiazzo 2020[7]	MedDRA	AE systematic assessment
Cohen 2020[8]	Not Specified	SF-36; AE systematic evaluation
Davies 2015[9]	MedDRA 15.1	AE systematic assessment
Davies 2021[10]	MedDRA 22	SF-36; IWQOL, AE systematic evaluation
Gadde 2011[11]	MedDRA 10.1 Suicidality C-SSRS Depression: PHQ-9> 10	PHQ-9, C-SSRS, AE systematic evaluation
Garvey 2020[12]	MedDRA 21	SF-36, IWQOL, AE systematic evaluation
Garvey 2022[13]	MedDRA 23	AE systematic evaluation
Garvey 2023[14]	MedDRA 25.1	Depress, suicid: PHQ-9, C-SSRS, AE systematic assessment, SF-36, IWQOL
Greenway 2010 [15]	MedDRA 9.1<br>Depression: IDS-SR >14	WQOL-Lite; IDS-SR; AE systematic assessment
Hollander 2013[16]	MedDRA 15.0 Depression: IDS-SR >14	Patient report
James 1997[17]	Not Specified	QOL questionnaire, patient report
Jastreboff 2022 [18]	MedDRA 27. Depression: PHQ-9 Suicidality C-SSRS	C-SSRS and PHQ-9, AE systematic evaluation
Kadowaki 2022 [19]	MedDRA 23	SF-36, IWQOL, systematic evaluation
Le Roux 2017[20]	MedDRA 15.1 Depression: PHQ-9 Suicidality C-SSRS	C-SSRS, PHQ-9, IWQOL, AE systematic evaluation
Lincoff 2023[21]	MedDRA 26	AE systematic evaluation
Loomba 2024[22]	MedDRA 26.1	AE systematic evaluation
Malhotra 2024[23]	MedDRA 26.1 Depression: PHQ-9 Suicidality C-SSRS	C-SSRS, PHQ-9, SF-36, EQ-5D-5, AE systematic evaluation
Mingrone 2021[24]	SF-36 Mental Health	SF-36, AE systematic evaluation
O'Neill 2018[25]	MedDRA 19 SF-36, IWQOL	SF-36, IWQOL, AE systematic evaluation
Rubino 2021[26]	MedDRA 23.1 Depression: PHQ-9 Suicidality C-SSRS	C-SSRS and PHQ-9, AE systematic evaluation
Schauer 2017[27]	MedRA, SF-36	SF-36, AE systematic evaluation
Wadden 2013[28]	MedDRA 22, SF-36	SF-36, AE systematic evaluation
Wadden 2021[29]	MedDRA 26.1 Depression: PHQ-9 Suicidality C-SSRS	C-SSRS, PHQ-9, SF-36, EQ-5D-5, AE systematic evaluation, IWQOL
Wilding 2021[30]	MedDRA 15.1 Depression: PHQ-9 Suicidality C-SSRS	C-SSRS, PHQ-9, SF-36 IWQOL, AE systematic evaluation

Table S3: Definitions and ascertainment methods of psychiatric adverse events across included trials. AE=Adverse event ESG= Endoscopic sleeve gastroplasty; LM= Lifestyle Modification; DJBL= Duodenal-Jejunal Bypass Liner; RYGB= Roux-en-Y Gastric Bypass; MedDRA= Medical Dictionary for Regulatory Activities; C-SSRS =Columbia Suicidality Severity Rating Scale; PHQ-9= Patient Health Questionnaire; WQOL= Impact of Weight on Quality of Life-Lite questionnaire. IDS-SR=Inventory of Depressive Symptomatology

Included Studies

[1] B. K. Abu Dayyeh *et al.*, 'Endoscopic sleeve gastroplasty for treatment of class 1 and 2 obesity (MERIT): a prospective, multicentre, randomised trial', *The Lancet*, vol. 400, no. 10350, pp. 441–451, Aug. 2022, doi: 10.1016/S0140-6736(22)01280-6.

[2] D. B. Allison *et al.*, 'Controlled-Release Phentermine/Topiramate in Severely Obese Adults: A Randomized Controlled Trial (EQUIP)', *Obesity*, vol. 20, no. 2, pp. 330–342, Feb. 2012, doi: 10.1038/oby.2011.330.

[3] C. M. Apovian *et al.*, 'A randomized, phase 3 trial of naltrexone SR/bupropion SR on weight and obesity-related risk factors (COR-II)', *Obesity*, vol. 21, no. 5, pp. 935–943, May 2013, doi: 10.1002/oby.20309.

[4] L. J. Aronne *et al.*, 'Continued Treatment With Tirzepatide for Maintenance of Weight Reduction in Adults With Obesity: The SURMOUNT-4 Randomized Clinical Trial', *JAMA*, vol. 331, no. 1, p. 38, Jan. 2024, doi: 10.1001/jama.2023.24945.

[5] on behalf of the NN8022-1807 Investigators *et al.*, 'Safety, tolerability and sustained weight loss over 2 years with the once-daily human GLP-1 analog, liraglutide', *Int J Obes*, vol. 36, no. 6, pp. 843–854, June 2012, doi: 10.1038/ijo.2011.158.

[6] H. Bliddal *et al.*, 'Once-Weekly Semaglutide in Persons with Obesity and Knee Osteoarthritis', *N Engl J Med*, vol. 391, no. 17, pp. 1573–1583, Oct. 2024, doi: 10.1056/NEJMoa2403664.

[7] R. Caiazzo *et al.*, 'Efficacy and Safety of the Duodeno-Jejunal Bypass Liner in Patients With Metabolic Syndrome: A Multicenter Randomized Controlled Trial (ENDOMETAB)', *Annals of Surgery*, vol. 272, no. 5, pp. 696–702, Nov. 2020, doi: 10.1097/SLA.0000000000004339.

[8] R. V. Cohen *et al.*, 'Effect of Gastric Bypass vs Best Medical Treatment on Early-Stage Chronic Kidney Disease in Patients With Type 2 Diabetes and Obesity: A Randomized Clinical Trial', *JAMA Surg*, vol. 155, no. 8, p. e200420, Aug. 2020, doi: 10.1001/jamasurg.2020.0420.

[9] M. J. Davies *et al.*, 'Efficacy of Liraglutide for Weight Loss Among Patients With Type 2 Diabetes: The SCALE Diabetes Randomized Clinical Trial', *JAMA*, vol. 314, no. 7, p. 687, Aug. 2015, doi: 10.1001/jama.2015.9676.

[10] M. Davies *et al.*, 'Semaglutide 2·4 mg once a week in adults with overweight or obesity, and type 2 diabetes (STEP 2): a randomised, double-blind, double-dummy, placebo-controlled, phase 3 trial', *The Lancet*, vol. 397, no. 10278, pp. 971–984, Mar. 2021, doi: 10.1016/S0140-6736(21)00213-0.

[11] K. M. Gadde *et al.*, 'Effects of low-dose, controlled-release, phentermine plus topiramate combination on weight and associated comorbidities in overweight and obese adults (CONQUER): a randomised, placebo-controlled, phase 3 trial', *The Lancet*, vol. 377, no. 9774, pp. 1341–1352, Apr. 2011, doi: 10.1016/S0140-6736(11)60205-5.

[12] W. T. Garvey *et al.*, 'Efficacy and Safety of Liraglutide 3.0 mg in Individuals With Overweight or Obesity and Type 2 Diabetes Treated With Basal Insulin: The SCALE Insulin Randomized Controlled Trial', *Diabetes Care*, vol. 43, no. 5, pp. 1085–1093, May 2020, doi: 10.2337/dc19-1745.

[13] W. T. Garvey *et al.*, 'Two-year effects of semaglutide in adults with overweight or obesity: the STEP 5 trial', *Nat Med*, vol. 28, no. 10, pp. 2083–2091, Oct. 2022, doi: 10.1038/s41591-022-02026-4.

[14] W. T. Garvey *et al.*, 'Tirzepatide once weekly for the treatment of obesity in people with type 2 diabetes (SURMOUNT-2): a double-blind, randomised, multicentre, placebo-controlled, phase 3 trial', *The Lancet*, vol. 402, no. 10402, pp. 613–626, Aug. 2023, doi: 10.1016/S0140-6736(23)01200-X.

[15] F. L. Greenway *et al.*, 'Effect of naltrexone plus bupropion on weight loss in overweight and obese adults (COR-I): a multicentre, randomised, double-blind, placebo-controlled, phase 3 trial', *The Lancet*, vol. 376, no. 9741, pp. 595–605, Aug. 2010, doi: 10.1016/S0140-6736(10)60888-4.

[16] P. Hollander *et al.*, 'Effects of Naltrexone Sustained- Release/Bupropion Sustained-Release Combination Therapy on Body Weight and Glycemic Parameters in Overweight and Obese Patients With Type 2 Diabetes', *Diabetes Care*, vol. 36, no. 12, pp. 4022–4029, Dec. 2013, doi: 10.2337/dc13-0234.

[17] W. P. James, A. Avenell, J. Broom, and J. Whitehead, 'A one-year trial to assess the value of orlistat in the management of obesity', *Int J Obes Relat Metab Disord*, vol. 21 Suppl 3, pp. S24-30, June 1997.

[18] A. M. Jastreboff *et al.*, 'Tirzepatide Once Weekly for the Treatment of Obesity', *N Engl J Med*, vol. 387, no. 3, pp. 205–216, July 2022, doi: 10.1056/NEJMoa2206038.

[19] T. Kadowaki *et al.*, 'Semaglutide once a week in adults with overweight or obesity, with or without type 2 diabetes in an east Asian population (STEP 6): a randomised, double-blind, double-dummy, placebo-controlled, phase 3a trial', *The Lancet Diabetes & Endocrinology*, vol. 10, no. 3, pp. 193–206, Mar. 2022, doi: 10.1016/S2213-8587(22)00008-0.

[20] C. W. Le Roux *et al.*, '3 years of liraglutide versus placebo for type 2 diabetes risk reduction and weight management in individuals with prediabetes: a randomised, double-blind trial', *The Lancet*, vol. 389, no. 10077, pp. 1399–1409, Apr. 2017, doi: 10.1016/S0140-6736(17)30069-7.

[21] A. M. Lincoff *et al.*, 'Semaglutide and Cardiovascular Outcomes in Obesity without Diabetes', *N Engl J Med*, vol. 389, no. 24, pp. 2221–2232, Dec. 2023, doi: 10.1056/NEJMoa2307563.

[22] R. Loomba *et al.*, 'Tirzepatide for Metabolic Dysfunction–Associated Steatohepatitis with Liver Fibrosis', *N Engl J Med*, vol. 391, no. 4, pp. 299–310, July 2024, doi: 10.1056/nejmoa2401943.

[23] A. Malhotra *et al.*, 'Tirzepatide for the Treatment of Obstructive Sleep Apnea and Obesity', *N Engl J Med*, vol. 391, no. 13, pp. 1193–1205, Oct. 2024, doi: 10.1056/NEJMoa2404881.

[24] G. Mingrone *et al.*, 'Metabolic surgery versus conventional medical therapy in patients with type 2 diabetes: 10-year follow-up of an open-label, single-centre, randomised controlled trial', *The Lancet*, vol. 397, no. 10271, pp. 293–304, Jan. 2021, doi: 10.1016/s0140-6736(20)32649-0.

[25] P. M. O'Neil *et al.*, 'Efficacy and safety of semaglutide compared with liraglutide and placebo for weight loss in patients with obesity: a randomised, double-blind, placebo and active controlled, dose-ranging, phase 2 trial', *The Lancet*, vol. 392, no. 10148, pp. 637–649, Aug. 2018, doi: 10.1016/s0140-6736(18)31773-2.

[26] D. M. Rubino *et al.*, 'Effect of Weekly Subcutaneous Semaglutide vs Daily Liraglutide on Body Weight in Adults With Overweight or Obesity Without Diabetes: The STEP 8 Randomized Clinical Trial', *JAMA*, vol. 327, no. 2, p. 138, Jan. 2022, doi: 10.1001/jama.2021.23619.

[27] P. R. Schauer *et al.*, 'Bariatric Surgery versus Intensive Medical Therapy for Diabetes — 5-Year Outcomes', *N Engl J Med*, vol. 376, no. 7, pp. 641–651, Feb. 2017, doi: 10.1056/NEJMoa1600869.

[28] on behalf of the NN8022-1923 Investigators *et al.*, 'Weight maintenance and additional weight loss with liraglutide after low-calorie-diet-induced weight loss: The SCALE Maintenance randomized study', *Int J Obes*, vol. 37, no. 11, pp. 1443–1451, Nov. 2013, doi: 10.1038/ijo.2013.120.

[29] T. A. Wadden *et al.*, 'Effect of Subcutaneous Semaglutide vs Placebo as an Adjunct to Intensive Behavioral Therapy on Body Weight in Adults With Overweight or Obesity: The STEP 3 Randomized Clinical Trial', *JAMA*, vol. 325, no. 14, p. 1403, Apr. 2021, doi: 10.1001/jama.2021.1831.

[30] J. P. H. Wilding *et al.*, 'Once-Weekly Semaglutide in Adults with Overweight or Obesity', *N Engl J Med*, vol. 384, no. 11, pp. 989–1002, Mar. 2021, doi: 10.1056/NEJMoa2032183.

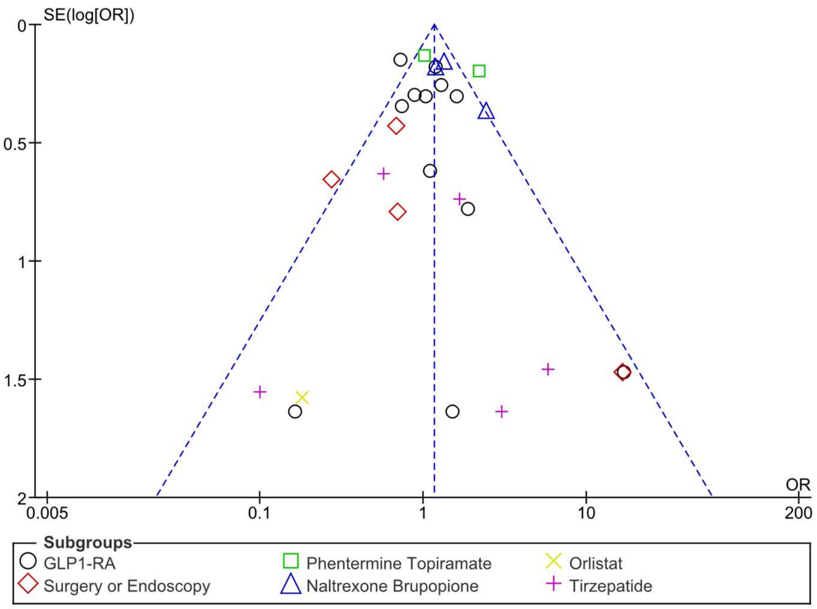

Figure S4: Funnel plot (Risk for serious and non-serious adverse events)

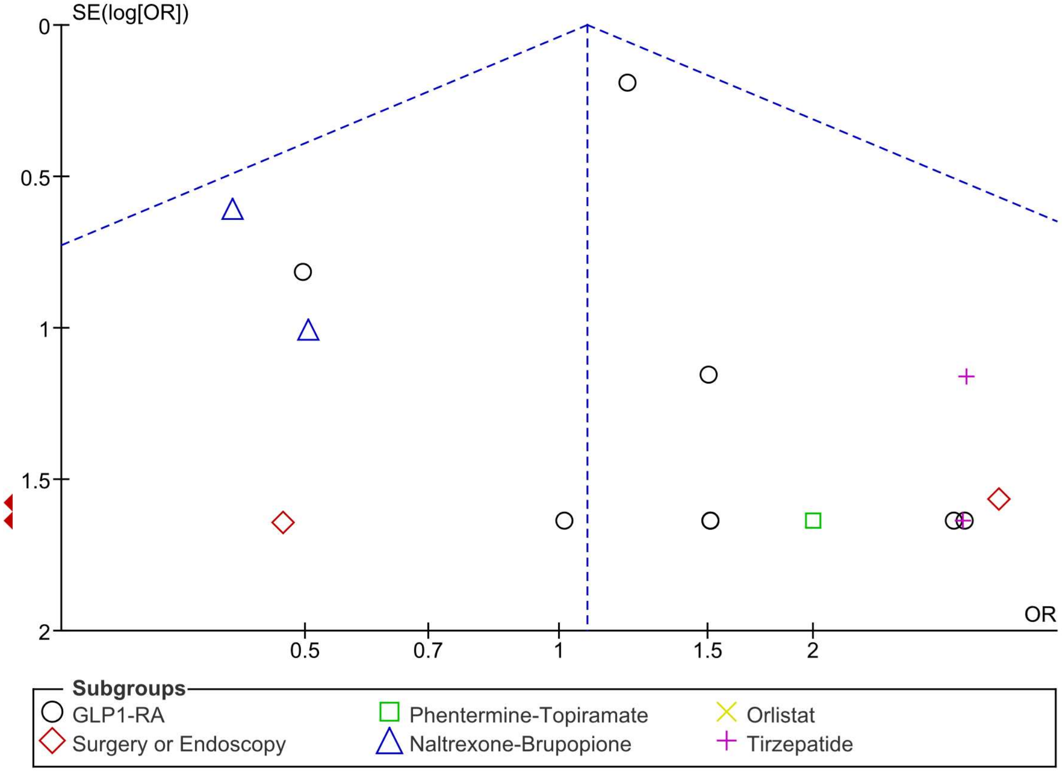

Figure S5: Funnel plot (Risk for serious adverse events)

Study or Subgroup	Weight Loss Events	Weight Loss Total	Control Events	Control Total	Weight	Odds Ratio M-H, Random, 95% CI	A	B	C	D	E	F
**10.27.1 GLP1-RA**												
Astrup 2012	7	93	0	98	0.5%	17.08 [0.96, 303.45]	+	?	?	?	?	?
Bliddal 2024	0	271	1	136	0.4%	0.17 [0.01, 4.11]	+	+	+	?	?	?
Davies 2015	1	423	0	212	0.4%	1.51 [0.06, 37.20]	+	+	+	+	?	?
Davies 2021	47	807	15	403	6.6%	1.60 [0.88, 2.90]	+	+	+	+	?	?
Garvey 2022	26	152	25	152	6.5%	1.05 [0.57, 1.91]	+	+	+	?	?	+
Leroux 2017	21	1505	14	749	5.6%	0.74 [0.38, 1.47]	+	+	+	+	+	+
Lincoff 2023	69	8803	58	8801	10.2%	1.19 [0.84, 1.69]	+	+	+	?	?	?
O' Neill 2018	5	103	6	136	2.4%	1.11 [0.33, 3.73]	+	+	+	+	?	+
Rubino 2021	11	253	2	85	1.6%	1.89 [0.41, 8.69]	+	?	?	+	?	?
Wadden 2013	24	212	26	206	6.6%	0.88 [0.49, 1.60]	+	+	+	+	?	?
Wadden 2021	60	407	24	204	7.7%	1.30 [0.78, 2.15]	+	+	+	+	+	+
Wilding 2021	124	1306	83	655	11.1%	0.72 [0.54, 0.97]	+	+	+	+	+	+
**Subtotal (95% CI)**		**14335**		**11837**	**59.5%**	**1.04 [0.83, 1.31]**						
Total events	395		254									
Heterogeneity: Tau² = 0.04; Chi² = 15.70, df = 11 (P = 0.15); I² = 30%												
Test for overall effect: Z = 0.34 (P = 0.74)												
**10.27.2 Surgery or Endoscopy**												
Abu Dayyeh 2022	3	77	14	110	2.2%	0.28 [0.08, 1.00]	+	?	?	?	?	?
Caiazzo 2020	10	49	0	31	0.5%	16.75 [0.94, 296.97]	?	?	?	?	?	?
Cohen 2020	3	51	4	49	1.6%	0.70 [0.15, 3.32]	+	?	?	+	+	?
Schauer 2017	19	99	11	43	4.2%	0.69 [0.30, 1.61]	+	?	?	+	?	?
**Subtotal (95% CI)**		**276**		**233**	**8.5%**	**0.76 [0.25, 2.30]**						
Total events	35		29									
Heterogeneity: Tau² = 0.68; Chi² = 7.08, df = 3 (P = 0.07); I² = 58%												
Test for overall effect: Z = 0.48 (P = 0.63)												
**10.27.4 Naltrexone-Bupropione**												
Apovian 2013	166	992	64	492	10.8%	1.34 [0.98, 1.83]	+	+	?	+	+	?
Greenway 2010	111	1142	47	569	10.0%	1.20 [0.84, 1.71]	+	+	+	+	+	+
Hollander 2013	44	333	10	169	5.3%	2.42 [1.19, 4.94]	+	?	+	+	+	?
**Subtotal (95% CI)**		**2467**		**1230**	**26.2%**	**1.40 [1.04, 1.87]**						
Total events	321		121									
Heterogeneity: Tau² = 0.02; Chi² = 3.02, df = 2 (P = 0.22); I² = 34%												
Test for overall effect: Z = 2.24 (P = 0.03)												
**10.27.5 Orlistat**												
James 1997	0	23	2	23	0.4%	0.18 [0.01, 4.03]	?	+	+	+	?	?
**Subtotal (95% CI)**		**23**		**23**	**0.4%**	**0.18 [0.01, 4.03]**						
Total events	0		2									
Heterogeneity: Not applicable												
Test for overall effect: Z = 1.08 (P = 0.28)												
**10.27.6 Tirzepatide**												
Aronne 2024	1	335	0	335	0.4%	3.01 [0.12, 74.13]	+	+	+	?	?	?
Astrup 2012	0	0	0	0		Not estimable	+	?	?	?	?	?
Garvey 2023	0	623	2	315	0.4%	0.10 [0.00, 2.10]	+	+	+	?	?	?
Jastreboff 2022	8	1896	0	643	0.5%	5.79 [0.33, 100.50]	+	+	+	+	?	?
Loomba 2024	6	95	5	48	2.3%	0.58 [0.17, 2.01]	+	+	+	?	?	?
Malhotra 2024	5	234	3	235	1.8%	1.69 [0.40, 7.15]	+	+	+	?	?	?
**Subtotal (95% CI)**		**3183**		**1576**	**5.5%**	**1.03 [0.36, 2.94]**						
Total events	20		10									
Heterogeneity: Tau² = 0.37; Chi² = 5.39, df = 4 (P = 0.25); I² = 26%												
Test for overall effect: Z = 0.06 (P = 0.95)												
**Total (95% CI)**		**20284**		**14899**	**100.0%**	**1.09 [0.89, 1.34]**						
Total events	771		416									
Heterogeneity: Tau² = 0.08; Chi² = 39.69, df = 24 (P = 0.02); I² = 40%												
Test for overall effect: Z = 0.82 (P = 0.41)												
Test for subgroup differences: Chi² = 4.32, df = 4 (P = 0.36), I² = 7.4%												

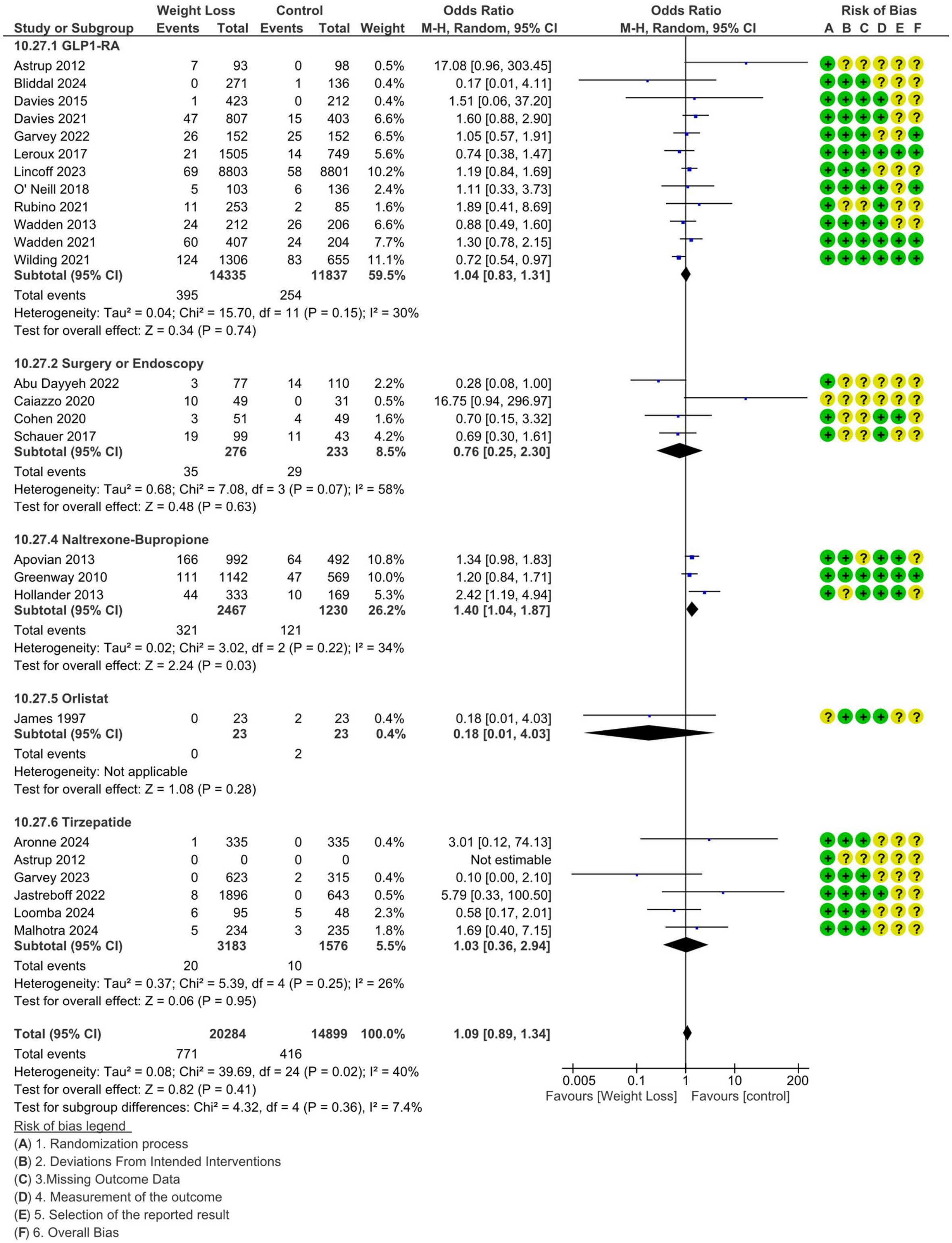

Risk of bias legend

(**A**) 1. Randomization process
(**B**) 2. Deviations From Intended Interventions
(**C**) 3.Missing Outcome Data
(**D**) 4. Measurement of the outcome
(**E**) 5. Selection of the reported result
(**F**) 6. Overall Bias

Fig. S6: Difference in risk in Psychiatric Serious and non-Serious Adverse Events between weight loss and controls EMA approved intervention analysis

Study or Subgroup	GLP-1RA Events	GLP-1RA Total	Control Events	Control Total	Weight	Odds Ratio M-H, Random, 95% CI	Risk of Bias A	B	C	D	E	F
**10.8.1 GLP1-RA**												
Astrup 2012	7	93	0	98	0.5%	17.08 [0.96, 303.45]	+	?	?	?	?	?
Bliddal 2024	0	271	1	136	0.4%	0.17 [0.01, 4.11]	+	+	+	?	?	?
Davies 2015	1	423	0	212	0.4%	1.51 [0.06, 37.20]	+	+	+	+	?	?
Davies 2021	47	807	15	403	5.5%	1.60 [0.88, 2.90]	+	+	+	+	?	?
Garvey 2022	26	152	25	152	5.4%	1.05 [0.57, 1.91]	+	+	+	?	?	+
Leroux 2017	21	1505	14	749	4.8%	0.74 [0.38, 1.47]	+	+	+	+	+	+
Lincoff 2023	69	8803	58	8801	8.1%	1.19 [0.84, 1.69]	+	+	+	?	?	?
O' Neill 2018	5	103	6	136	2.1%	1.11 [0.33, 3.73]	+	+	+	+	?	+
Rubino 2021	11	253	2	85	1.5%	1.89 [0.41, 8.69]	+	?	?	+	?	?
Wadden 2013	24	212	26	206	5.5%	0.88 [0.49, 1.60]	+	+	+	+	?	?
Wadden 2021	60	407	24	204	6.4%	1.30 [0.78, 2.15]	+	+	+	+	+	+
Wilding 2021	124	1306	83	655	8.8%	0.72 [0.54, 0.97]	+	+	+	+	+	+
**Subtotal (95% CI)**		**14335**		**11837**	**49.3%**	**1.04 [0.83, 1.31]**						
Total events	395		254									
Heterogeneity: Tau² = 0.04; Chi² = 15.70, df = 11 (P = 0.15); I² = 30%												
Test for overall effect: Z = 0.34 (P = 0.74)												
**10.8.2 Surgery**												
Abu Dayyeh 2022	3	77	14	110	2.0%	0.28 [0.08, 1.00]	+	?	?	?	?	?
Caiazzo 2020	10	49	0	31	0.5%	16.75 [0.94, 296.97]	?	?	?	?	?	?
Cohen 2020	3	51	4	49	1.4%	0.70 [0.15, 3.32]	+	?	?	+	+	?
Schauer 2017	19	99	11	43	3.6%	0.69 [0.30, 1.61]	+	?	?	+	?	?
**Subtotal (95% CI)**		**276**		**233**	**7.5%**	**0.76 [0.25, 2.30]**						
Total events	35		29									
Heterogeneity: Tau² = 0.68; Chi² = 7.08, df = 3 (P = 0.07); I² = 58%												
Test for overall effect: Z = 0.48 (P = 0.63)												
**10.8.3 Phent/TOP**												
Allison 2012	110	751	37	513	7.7%	2.21 [1.49, 3.26]	+	+	?	+	+	?
Gadde 2011	155	1492	101	993	9.1%	1.02 [0.79, 1.33]	+	+	+	+	+	+
**Subtotal (95% CI)**		**2243**		**1506**	**16.8%**	**1.48 [0.70, 3.15]**						
Total events	265		138									
Heterogeneity: Tau² = 0.27; Chi² = 10.21, df = 1 (P = 0.001); I² = 90%												
Test for overall effect: Z = 1.02 (P = 0.31)												
**10.8.4 Naltr Brup**												
Apovian 2013	166	992	64	492	8.6%	1.34 [0.98, 1.83]	+	+	?	+	+	?
Greenway 2010	111	1142	47	569	8.1%	1.20 [0.84, 1.71]	+	+	+	+	+	+
Hollander 2013	44	333	10	169	4.5%	2.42 [1.19, 4.94]	+	?	+	+	+	?
**Subtotal (95% CI)**		**2467**		**1230**	**21.2%**	**1.40 [1.04, 1.87]**						
Total events	321		121									
Heterogeneity: Tau² = 0.02; Chi² = 3.02, df = 2 (P = 0.22); I² = 34%												
Test for overall effect: Z = 2.24 (P = 0.03)												
**10.8.5 Orlistat**												
James 1997	0	23	2	23	0.4%	0.18 [0.01, 4.03]	?	+	+	+	?	?
**Subtotal (95% CI)**		**23**		**23**	**0.4%**	**0.18 [0.01, 4.03]**						
Total events	0		2									
Heterogeneity: Not applicable												
Test for overall effect: Z = 1.08 (P = 0.28)												
**10.8.6 Tirzepatide**												
Aronne 2024	1	335	0	335	0.4%	3.01 [0.12, 74.13]	+	+	+	?	?	?
Astrup 2012	0	0	0	0		Not estimable	+	?	?	?	?	?
Garvey 2023	0	623	2	315	0.4%	0.10 [0.00, 2.10]	+	+	+	?	?	?
Jastreboff 2022	8	1896	0	643	0.5%	5.79 [0.33, 100.50]	+	+	+	+	?	?
Loomba 2024	6	95	5	48	2.1%	0.58 [0.17, 2.01]	+	+	+	?	?	?
Malhotra 2024	5	234	3	235	1.6%	1.69 [0.40, 7.15]	+	+	+	?	?	?
**Subtotal (95% CI)**		**3183**		**1576**	**4.9%**	**1.03 [0.36, 2.94]**						
Total events	20		10									
Heterogeneity: Tau² = 0.37; Chi² = 5.39, df = 4 (P = 0.25); I² = 26%												
Test for overall effect: Z = 0.06 (P = 0.95)												
**Total (95% CI)**		**22527**		**16405**	**100.0%**	**1.14 [0.94, 1.39]**						
Total events	1036		554									
Heterogeneity: Tau² = 0.09; Chi² = 51.98, df = 26 (P = 0.002); I² = 50%												
Test for overall effect: Z = 1.33 (P = 0.18)												
Test for subgroup differences: Chi² = 4.76, df = 5 (P = 0.45), I² = 0%												

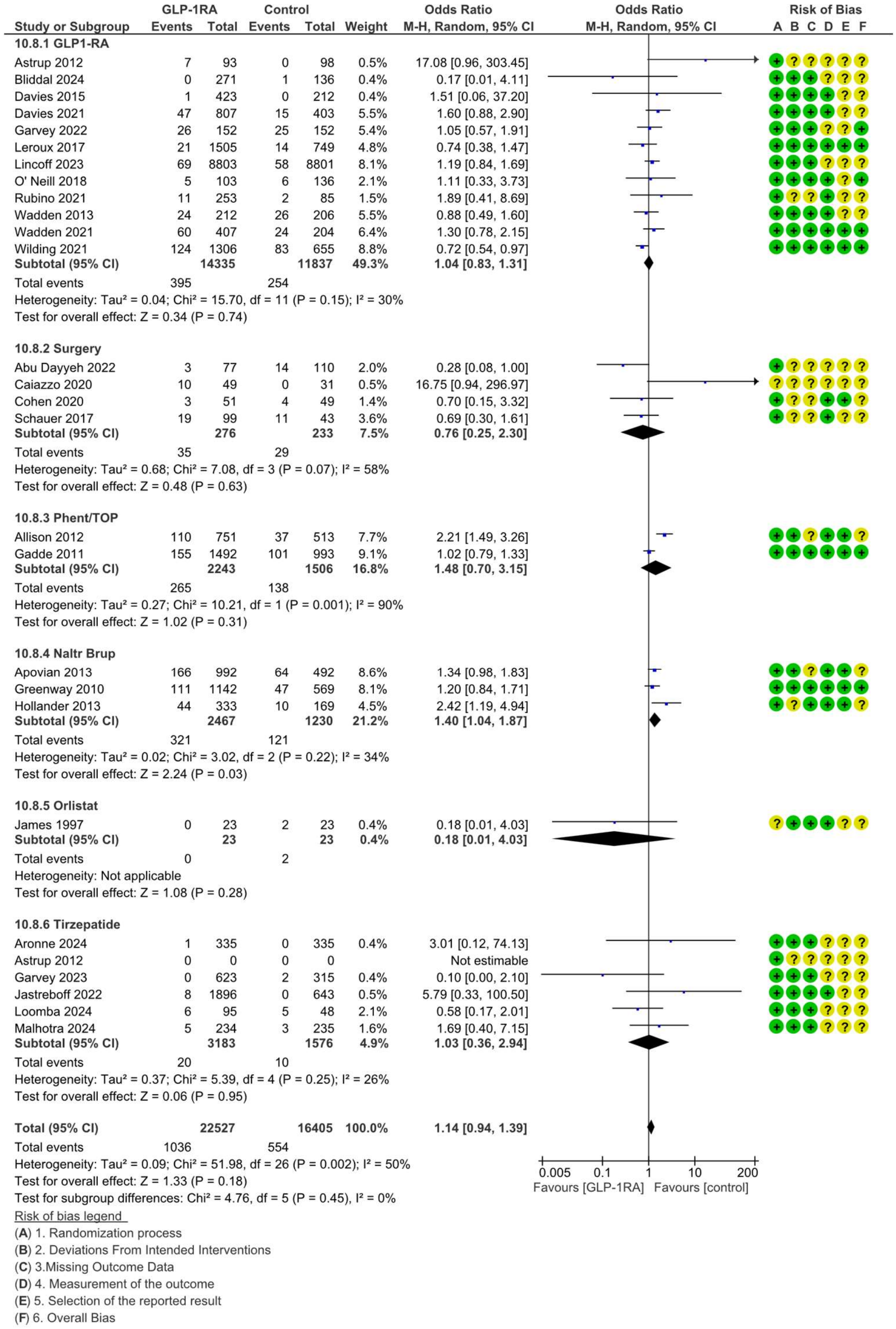

Risk of bias legend

(**A**) 1. Randomization process
(**B**) 2. Deviations From Intended Interventions
(**C**) 3.Missing Outcome Data
(**D**) 4. Measurement of the outcome
(**E**) 5. Selection of the reported result
(**F**) 6. Overall Bias

Fig. S7: Difference in risk in Psychiatric Serious and non-Serious Adverse Events between weight reducing therapies (EMA and FDA approved intervention analysis), and controls

Study or Subgroup	Weight Loss Events	Weight Loss Total	Control Events	Control Total	Weight	Odds Ratio M-H, Fixed, 95% CI
**10.6.1 GLP1-RA**						
Bliddal 2024	0	271	1	136	2.6%	0.17 [0.01, 4.11]
Davies 2015	1	423	0	212	0.9%	1.51 [0.06, 37.20]
Garvey 2022	1	152	0	152	0.7%	3.02 [0.12, 74.72]
Leroux 2017	3	1505	3	749	5.3%	0.50 [0.10, 2.47]
Lincoff 2023	59	8803	49	8801	64.0%	1.21 [0.82, 1.76]
Rubino 2021	1	253	0	85	1.0%	1.02 [0.04, 25.17]
Wadden 2013	1	212	0	206	0.7%	2.93 [0.12, 72.32]
Wadden 2021	1	407	0	204	0.9%	1.51 [0.06, 37.21]
Wilding 2021	3	1306	1	655	1.7%	1.51 [0.16, 14.50]
**Subtotal (95% CI)**		**13332**		**11200**	**77.7%**	**1.16 [0.82, 1.65]**
Total events	70		54			
Heterogeneity: Chi² = 3.29, df = 8 (P = 0.91); I² = 0%						
Test for overall effect: Z = 0.85 (P = 0.39)						
**10.6.2 Surgery or Endoscopy**						
Abu Dayyeh 2022	0	77	1	110	1.6%	0.47 [0.02, 11.71]
Caiazzo 2020	2	49	0	31	0.8%	3.32 [0.15, 71.40]
**Subtotal (95% CI)**		**126**		**141**	**2.4%**	**1.38 [0.20, 9.38]**
Total events	2		1			
Heterogeneity: Chi² = 0.74, df = 1 (P = 0.39); I² = 0%						
Test for overall effect: Z = 0.33 (P = 0.74)						
**10.6.3 Phentermine-Topiramate**						
Gadde 2011	1	1492	0	993	0.8%	2.00 [0.08, 49.10]
**Subtotal (95% CI)**		**1492**		**993**	**0.8%**	**2.00 [0.08, 49.10]**
Total events	1		0			
Heterogeneity: Not applicable						
Test for overall effect: Z = 0.42 (P = 0.67)						
**10.6.4 Naltrexone-Brupopione**						
Apovian 2013	5	992	6	492	10.5%	0.41 [0.12, 1.35]
Hollander 2013	2	333	2	169	3.5%	0.50 [0.07, 3.61]
**Subtotal (95% CI)**		**1325**		**661**	**14.0%**	**0.43 [0.16, 1.20]**
Total events	7		8			
Heterogeneity: Chi² = 0.03, df = 1 (P = 0.86); I² = 0%						
Test for overall effect: Z = 1.61 (P = 0.11)						
**10.6.5 Orlistat**						
James 1997	0	23	2	23	3.2%	0.18 [0.01, 4.03]
**Subtotal (95% CI)**		**23**		**23**	**3.2%**	**0.18 [0.01, 4.03]**
Total events	0		2			
Heterogeneity: Not applicable						
Test for overall effect: Z = 1.08 (P = 0.28)						
**10.6.6 Tirzepatide**						
Aronne 2024	1	335	0	335	0.7%	3.01 [0.12, 74.13]
Malhotra 2024	3	234	1	235	1.3%	3.04 [0.31, 29.43]
**Subtotal (95% CI)**		**569**		**570**	**2.0%**	**3.03 [0.48, 19.31]**
Total events	4		1			
Heterogeneity: Chi² = 0.00, df = 1 (P = 1.00); I² = 0%						
Test for overall effect: Z = 1.17 (P = 0.24)						
**Total (95% CI)**		**16867**		**13588**	**100.0%**	**1.08 [0.79, 1.47]**
Total events	84		66			
Heterogeneity: Chi² = 9.94, df = 16 (P = 0.87); I² = 0%						
Test for overall effect: Z = 0.47 (P = 0.64)						
Test for subgroup differences: Chi² = 5.92, df = 5 (P = 0.31), I² = 15.5%						

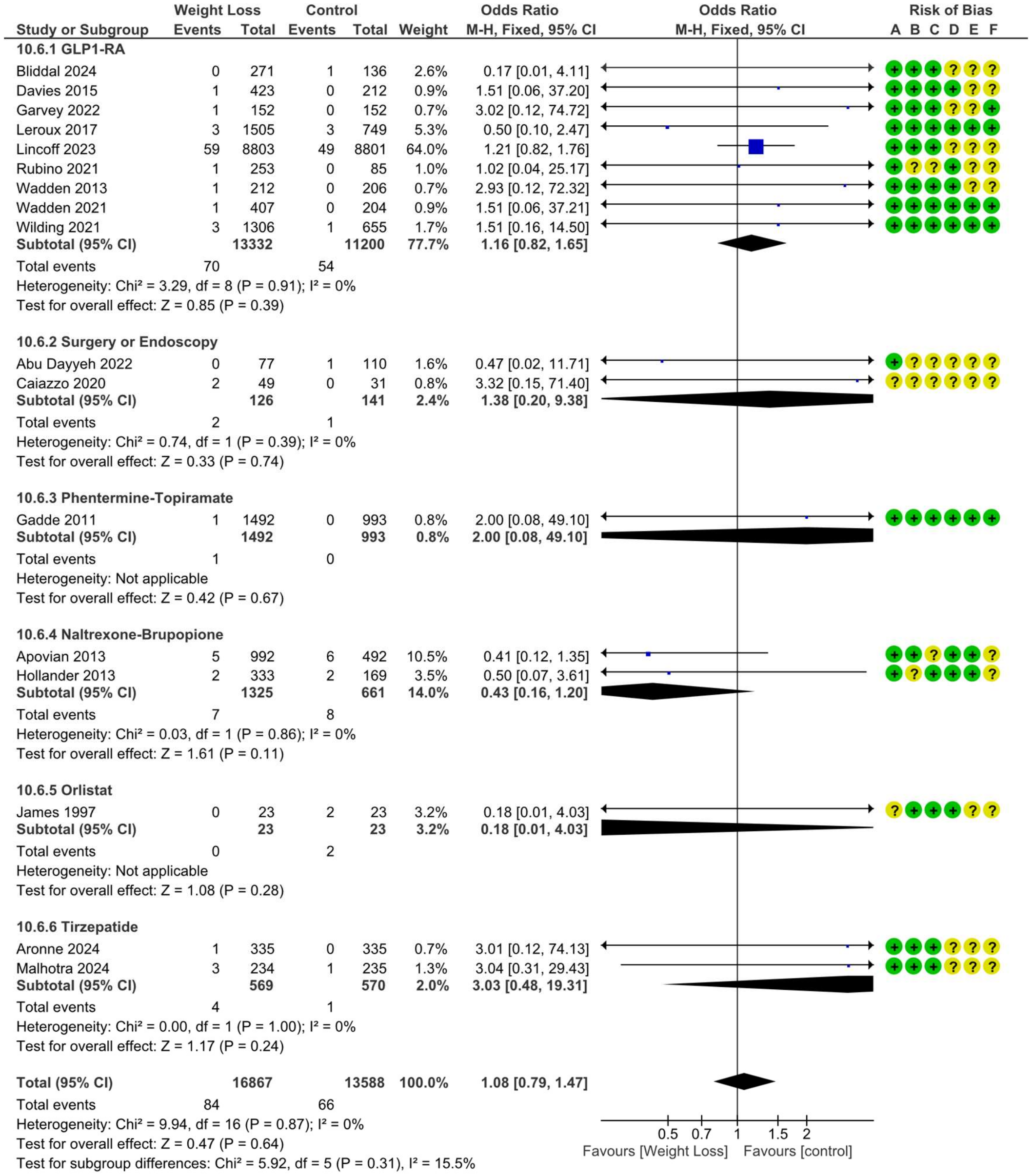

Risk of bias legend
(**A**) 1. Randomization process
(**B**) 2. Deviations From Intended Interventions
(**C**) 3.Missing Outcome Data
(**D**) 4. Measurement of the outcome
(**E**) 5. Selection of the reported result
(**F**) 6. Overall Bias

Fig. S8: Difference in risk in Psychiatric Serious Adverse Events between weight loss and controls (only EMA approved intervention analysis)

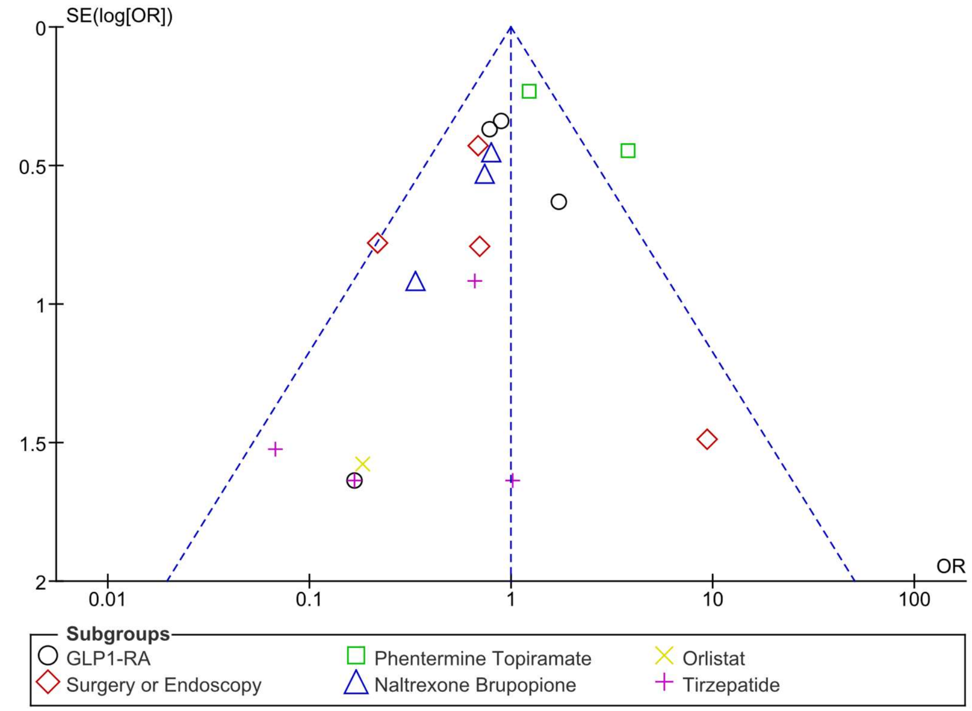

Figure S9: Funnel plot (Incidence of depression)

Study or Subgroup	GLP-1RA Events	GLP-1RA Total	Control Events	Control Total	Weight	Odds Ratio M-H, Fixed, 95% CI	A	B	C	D	E	F
**10.2.1 GLP1-RA**												
Bliddal 2024	0	271	1	136	2.0%	0.17 [0.01, 4.11]	+	+	+	?	?	?
Leroux 2017	19	1505	12	749	15.6%	0.79 [0.38, 1.63]	+	+	+	+	+	+
Lincoff 2023	16	8803	18	8801	17.7%	0.89 [0.45, 1.74]	+	+	+	?	?	?
Wadden 2013	7	212	4	206	3.9%	1.72 [0.50, 5.98]	+	+	+	+	?	?
**Subtotal (95% CI)**		**10791**		**9892**	**39.2%**	**0.89 [0.57, 1.40]**						
Total events	42		35									
Heterogeneity: Chi² = 2.25, df = 3 (P = 0.52); I² = 0%												
Test for overall effect: Z = 0.49 (P = 0.63)												
**10.2.2 Surgery or Endoscopy**												
Abu Dayyeh 2022	2	77	12	110	9.5%	0.22 [0.05, 1.00]	+	?	?	?	?	?
Caiazzo 2020	6	49	0	31	0.5%	9.41 [0.51, 173.28]	?	?	?	?	?	?
Cohen 2020	3	51	4	49	3.8%	0.70 [0.15, 3.32]	+	?	?	+	+	?
Schauer 2017	19	99	11	43	12.2%	0.69 [0.30, 1.61]	+	?	?	+	?	?
**Subtotal (95% CI)**		**276**		**233**	**26.0%**	**0.70 [0.39, 1.25]**						
Total events	30		27									
Heterogeneity: Chi² = 5.30, df = 3 (P = 0.15); I² = 43%												
Test for overall effect: Z = 1.21 (P = 0.23)												
**10.2.4 Naltrexone-Bupropione**												
Apovian 2013	13	992	8	492	10.4%	0.80 [0.33, 1.95]	+	+	?	+	+	?
Greenway 2010	9	1142	6	569	7.8%	0.75 [0.26, 2.10]	+	+	+	+	+	+
Hollander 2013	2	333	3	169	3.9%	0.33 [0.06, 2.02]	+	?	+	+	+	?
**Subtotal (95% CI)**		**2467**		**1230**	**22.2%**	**0.70 [0.37, 1.31]**						
Total events	24		17									
Heterogeneity: Chi² = 0.75, df = 2 (P = 0.69); I² = 0%												
Test for overall effect: Z = 1.12 (P = 0.26)												
**10.2.5 Orlistat**												
James 1997	0	23	2	23	2.4%	0.18 [0.01, 4.03]	?	+	+	+	?	?
**Subtotal (95% CI)**		**23**		**23**	**2.4%**	**0.18 [0.01, 4.03]**						
Total events	0		2									
Heterogeneity: Not applicable												
Test for overall effect: Z = 1.08 (P = 0.28)												
**10.2.6 Tirzepatide**												
Garvey 2023	0	623	1	315	2.0%	0.17 [0.01, 4.14]	+	+	+	?	?	?
Jastreboff 2022	1	1896	0	643	0.7%	1.02 [0.04, 25.03]	+	+	+	+	?	?
Loomba 2024	0	95	3	48	4.6%	0.07 [0.00, 1.35]	+	+	+	?	?	?
Malhotra 2024	2	234	3	235	2.9%	0.67 [0.11, 4.03]	+	+	+	?	?	?
**Subtotal (95% CI)**		**2848**		**1241**	**10.2%**	**0.33 [0.10, 1.04]**						
Total events	3		7									
Heterogeneity: Chi² = 2.31, df = 3 (P = 0.51); I² = 0%												
Test for overall effect: Z = 1.89 (P = 0.06)												
**Total (95% CI)**		**16405**		**12619**	**100.0%**	**0.72 [0.54, 0.97]**						
Total events	99		88									
Heterogeneity: Chi² = 13.24, df = 15 (P = 0.58); I² = 0%												
Test for overall effect: Z = 2.13 (P = 0.03)												
Test for subgroup differences: Chi² = 3.42, df = 4 (P = 0.49), I² = 0%												

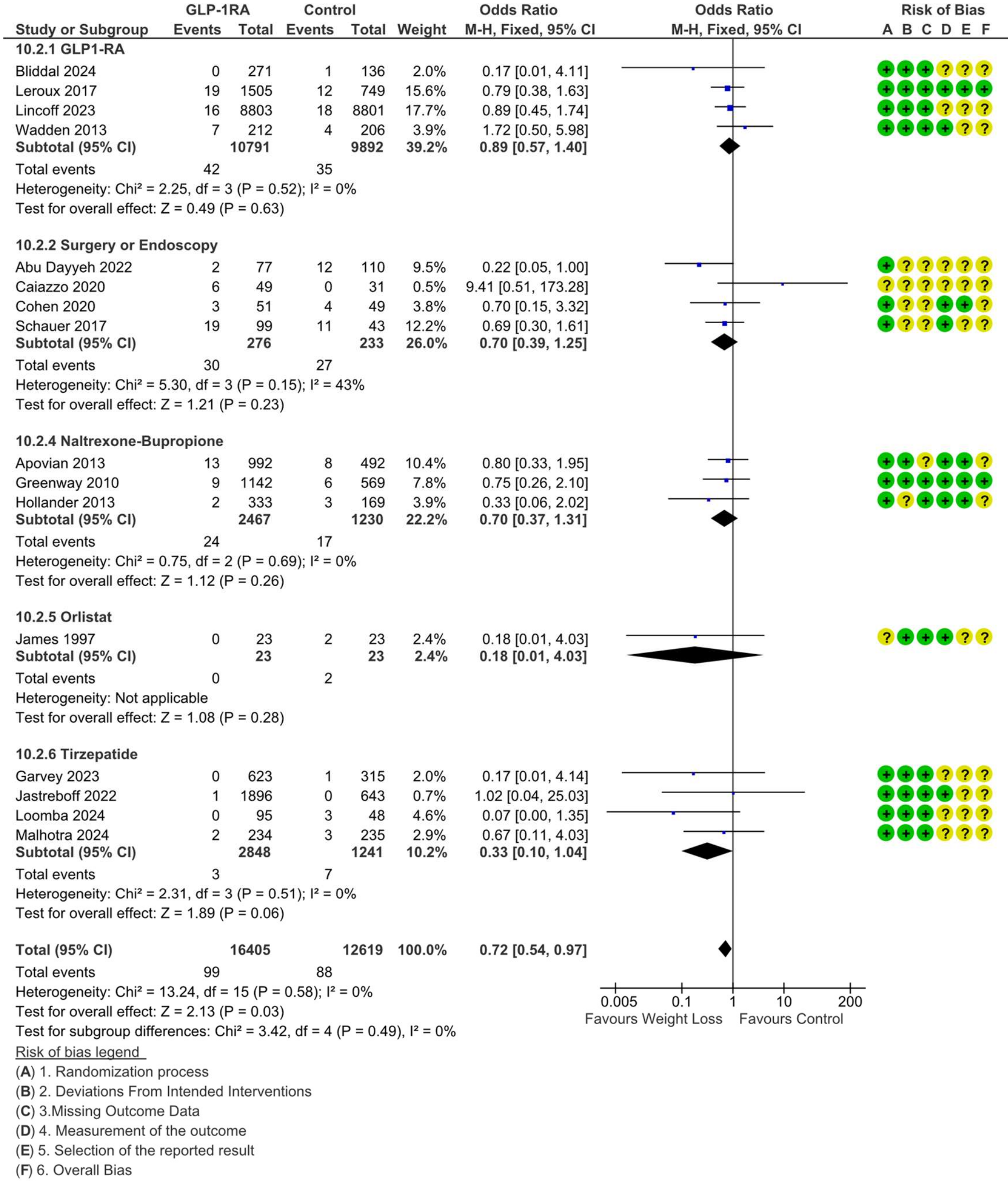

Risk of bias legend

(**A**) 1. Randomization process
(**B**) 2. Deviations From Intended Interventions
(**C**) 3.Missing Outcome Data
(**D**) 4. Measurement of the outcome
(**E**) 5. Selection of the reported result
(**F**) 6. Overall Bias

Figure S10 Incidence of depression, subgroup analysis by intervention (EMA-approved interventions)

Study or Subgroup	GLP-1RA Events	GLP-1RA Total	Control Events	Control Total	Weight	Odds Ratio M-H, Fixed, 95% CI	A	B	C	D	E	F
**10.26.2 Baseline BMI ≤ 40**												
Apovian 2013	13	992	8	492	10.4%	0.80 [0.33, 1.95]	+	+	?	+	+	?
Garvey 2023	0	623	1	315	2.0%	0.17 [0.01, 4.14]	+	+	+	?	?	?
Greenway 2010	9	1142	6	569	7.8%	0.75 [0.26, 2.10]	+	+	+	+	+	+
Hollander 2013	2	333	3	169	3.9%	0.33 [0.06, 2.02]	+	?	+	+	+	?
James 1997	0	23	2	23	2.4%	0.18 [0.01, 4.03]	?	+	+	+	?	?
Jastreboff 2022	1	1896	0	643	0.7%	1.02 [0.04, 25.03]	+	+	+	+	?	?
Leroux 2017	19	1505	12	749	15.6%	0.79 [0.38, 1.63]	+	+	+	+	+	+
Lincoff 2023	16	8803	18	8801	17.7%	0.89 [0.45, 1.74]	+	+	+	?	?	?
Loomba 2024	0	95	3	48	4.6%	0.07 [0.00, 1.35]	+	+	+	?	?	?
Malhotra 2024	2	234	3	235	2.9%	0.67 [0.11, 4.03]	+	+	+	?	?	?
Wadden 2013	7	212	4	206	3.9%	1.72 [0.50, 5.98]	+	+	+	+	?	?
**Subtotal (95% CI)**		**15858**		**12250**	**72.0%**	**0.75 [0.53, 1.06]**						
Total events	69		60									
Heterogeneity: Chi² = 6.95, df = 10 (P = 0.73); I² = 0%												
Test for overall effect: Z = 1.63 (P = 0.10)												
**10.26.6 Baseline BMI >40**												
Abu Dayyeh 2022	2	77	12	110	9.5%	0.22 [0.05, 1.00]	+	?	?	?	?	?
Bliddal 2024	0	271	1	136	2.0%	0.17 [0.01, 4.11]	+	+	+	?	?	?
Caiazzo 2020	6	49	0	31	0.5%	9.41 [0.51, 173.28]	?	?	?	?	?	?
Cohen 2020	3	51	4	49	3.8%	0.70 [0.15, 3.32]	+	?	?	+	+	?
Schauer 2017	19	99	11	43	12.2%	0.69 [0.30, 1.61]	+	?	?	+	?	?
**Subtotal (95% CI)**		**547**		**369**	**28.0%**	**0.66 [0.37, 1.17]**						
Total events	30		28									
Heterogeneity: Chi² = 5.95, df = 4 (P = 0.20); I² = 33%												
Test for overall effect: Z = 1.42 (P = 0.15)												
**Total (95% CI)**		**16405**		**12619**	**100.0%**	**0.72 [0.54, 0.97]**						
Total events	99		88									
Heterogeneity: Chi² = 13.24, df = 15 (P = 0.58); I² = 0%												
Test for overall effect: Z = 2.13 (P = 0.03)												
Test for subgroup differences: Chi² = 0.15, df = 1 (P = 0.70), I² = 0%												

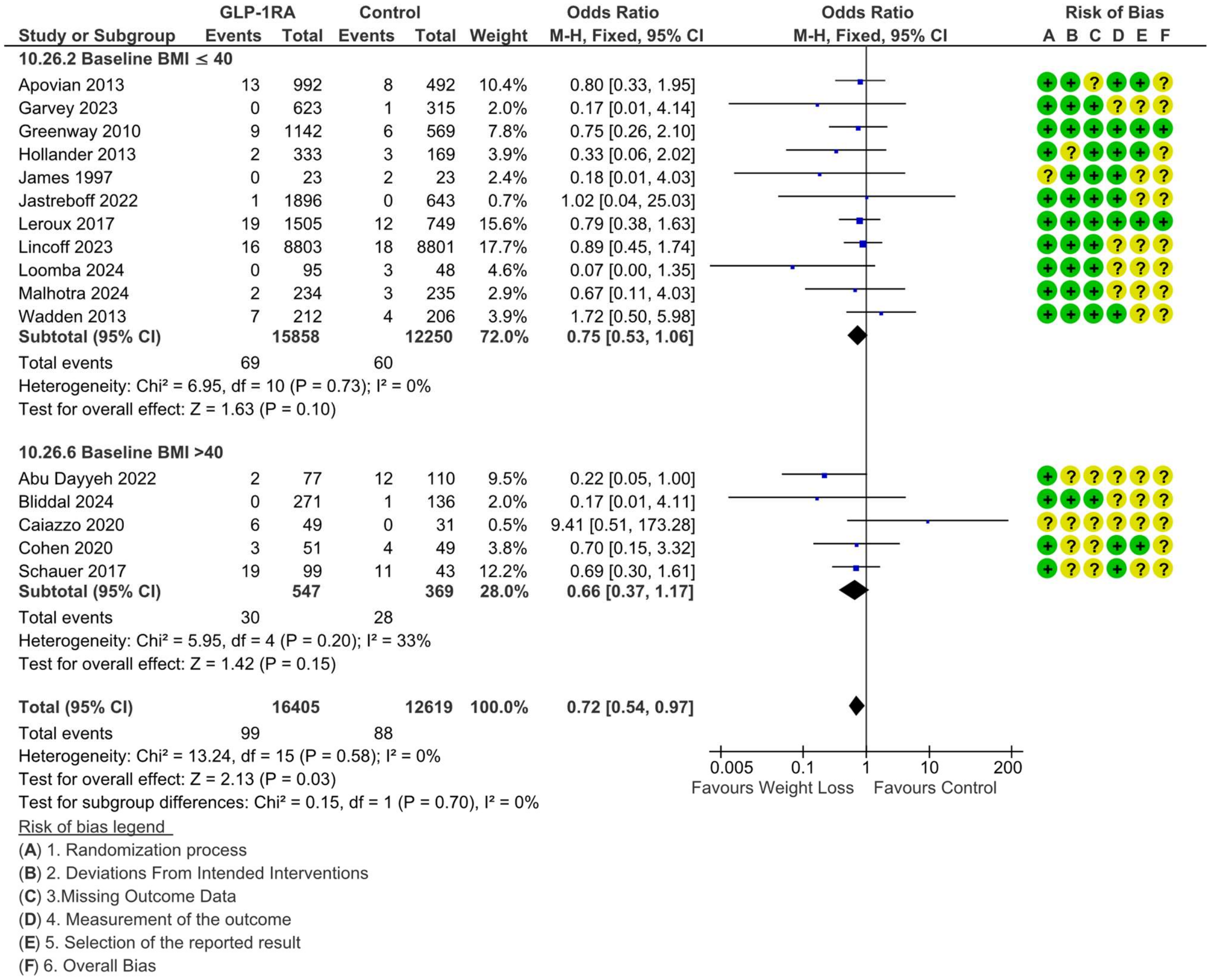

Risk of bias legend

(**A**) 1. Randomization process
(**B**) 2. Deviations From Intended Interventions
(**C**) 3.Missing Outcome Data
(**D**) 4. Measurement of the outcome
(**E**) 5. Selection of the reported result
(**F**) 6. Overall Bias

Fig. S11: Difference in risk in Depression between weight loss and controls, subgroup analysis by baseline BMI; EMA Analysis

Study or Subgroup	Weight loss Events	Weight loss Total	Control Events	Control Total	Weight	Odds Ratio M-H, Random, 95% CI	A	B	C	D	E	F
**10.2.1 GLP1-RA**												
Wadden 2013	7	212	4	206	5.8%	1.72 [0.50, 5.98]	+	+	+	+	?	?
Lincoff 2023	16	8803	18	8801	11.5%	0.89 [0.45, 1.74]	+	+	+	?	?	?
Leroux 2017	19	1505	12	749	10.8%	0.79 [0.38, 1.63]	+	+	+	+	+	+
Bliddal 2024	0	271	1	136	1.2%	0.17 [0.01, 4.11]	+	+	+	?	?	?
**Subtotal (95% CI)**		**10791**		**9892**	**29.3%**	**0.89 [0.57, 1.41]**						
Total events	42		35									
Heterogeneity: Tau² = 0.00; Chi² = 2.25, df = 3 (P = 0.52); I² = 0%												
Test for overall effect: Z = 0.48 (P = 0.63)												
**10.2.2 Surgery or Endoscopy**												
Schauer 2017	19	99	11	43	9.3%	0.69 [0.30, 1.61]	+	?	?	+	?	?
Cohen 2020	3	51	4	49	4.2%	0.70 [0.15, 3.32]	+	?	?	+	+	?
Caiazzo 2020	6	49	0	31	1.4%	9.41 [0.51, 173.28]	?	?	?	?	?	?
Abu Dayyeh 2022	2	77	12	110	4.3%	0.22 [0.05, 1.00]	+	?	?	?	?	?
**Subtotal (95% CI)**		**276**		**233**	**19.2%**	**0.67 [0.25, 1.82]**						
Total events	30		27									
Heterogeneity: Tau² = 0.44; Chi² = 5.30, df = 3 (P = 0.15); I² = 43%												
Test for overall effect: Z = 0.78 (P = 0.43)												
**10.2.3 Phentermine Topiramate**												
Gadde 2011	53	1492	29	993	14.6%	1.22 [0.77, 1.94]	+	+	+	+	+	+
Allison 2012	32	751	6	513	9.0%	3.76 [1.56, 9.06]	+	+	?	+	+	?
**Subtotal (95% CI)**		**2243**		**1506**	**23.6%**	**2.01 [0.67, 6.06]**						
Total events	85		35									
Heterogeneity: Tau² = 0.51; Chi² = 4.99, df = 1 (P = 0.03); I² = 80%												
Test for overall effect: Z = 1.24 (P = 0.21)												
**10.2.4 Naltrexone Brupopione**												
Hollander 2013	2	333	3	169	3.3%	0.33 [0.06, 2.02]	+	?	+	+	+	?
Greenway 2010	9	1142	6	569	7.4%	0.75 [0.26, 2.10]	+	+	+	+	+	+
Apovian 2013	13	992	8	492	8.9%	0.80 [0.33, 1.95]	+	+	?	+	+	?
**Subtotal (95% CI)**		**2467**		**1230**	**19.6%**	**0.70 [0.37, 1.32]**						
Total events	24		17									
Heterogeneity: Tau² = 0.00; Chi² = 0.75, df = 2 (P = 0.69); I² = 0%												
Test for overall effect: Z = 1.10 (P = 0.27)												
**10.2.5 Orlistat**												
James 1997	0	23	2	23	1.3%	0.18 [0.01, 4.03]	?	+	+	+	?	?
**Subtotal (95% CI)**		**23**		**23**	**1.3%**	**0.18 [0.01, 4.03]**						
Total events	0		2									
Heterogeneity: Not applicable												
Test for overall effect: Z = 1.08 (P = 0.28)												
**10.2.6 Tirzepatide**												
Malhotra 2024	2	234	3	235	3.3%	0.67 [0.11, 4.03]	+	+	+	?	?	?
Loomba 2024	0	95	3	48	1.4%	0.07 [0.00, 1.35]	+	+	+	?	?	?
Jastreboff 2022	1	1896	0	643	1.2%	1.02 [0.04, 25.03]	+	+	+	+	?	?
Garvey 2023	0	623	1	315	1.2%	0.17 [0.01, 4.14]	+	+	+	?	?	?
**Subtotal (95% CI)**		**2848**		**1241**	**7.1%**	**0.38 [0.11, 1.35]**						
Total events	3		7									
Heterogeneity: Tau² = 0.00; Chi² = 2.31, df = 3 (P = 0.51); I² = 0%												
Test for overall effect: Z = 1.50 (P = 0.13)												
**Total (95% CI)**		**18648**		**14125**	**100.0%**	**0.87 [0.61, 1.25]**						
Total events	184		123									
Heterogeneity: Tau² = 0.18; Chi² = 26.56, df = 17 (P = 0.06); I² = 36%												
Test for overall effect: Z = 0.73 (P = 0.47)												
Test for subgroup differences: Chi² = 5.40, df = 5 (P = 0.37), I² = 7.4%												

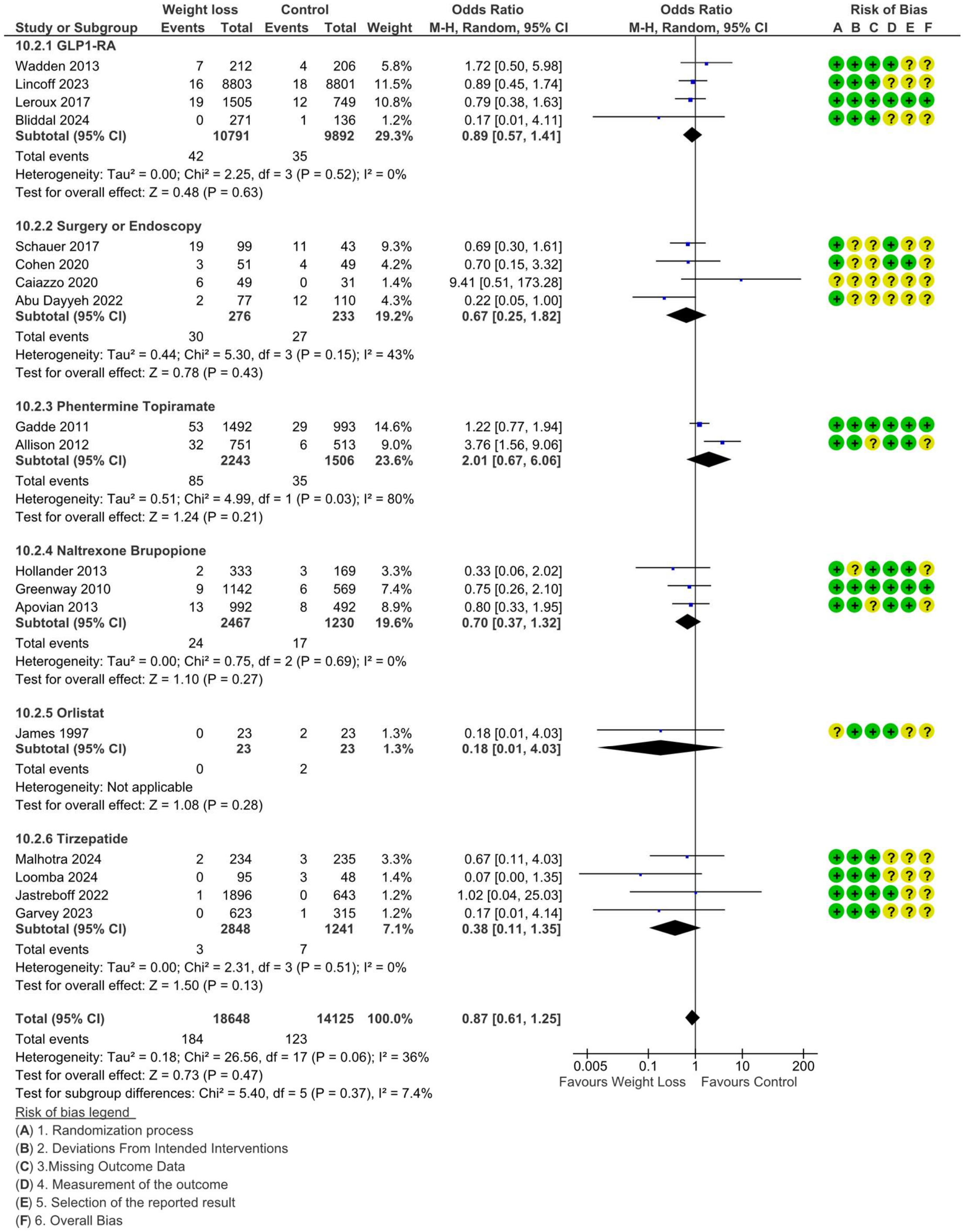

Risk of bias legend

(**A**) 1. Randomization process
(**B**) 2. Deviations From Intended Interventions
(**C**) 3.Missing Outcome Data
(**D**) 4. Measurement of the outcome
(**E**) 5. Selection of the reported result
(**F**) 6. Overall Bias

Figure S12: Incidence of depression, subgroup analysis by intervention (EMA-and FDA approved interventions)

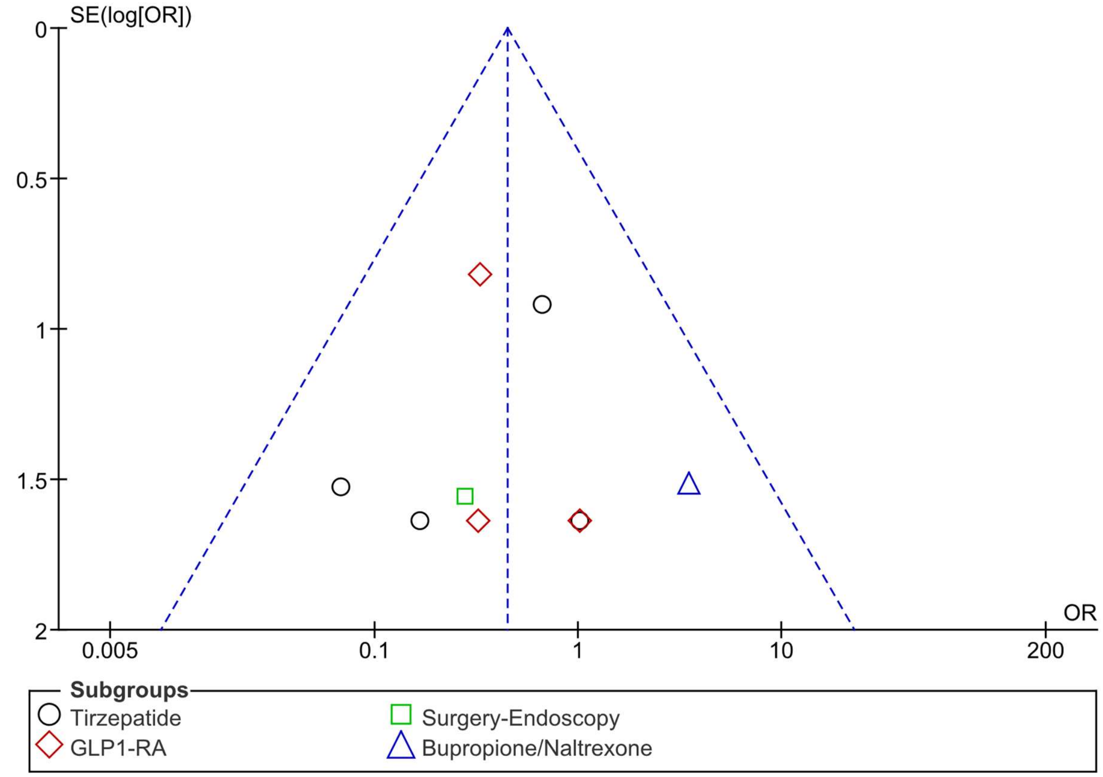

Figure S13:: Funnel plot (Risk for Major Depression)

Study or Subgroup	Weight Loss Events	Weight Loss Total	Control Events	Control Total	Weight	Odds Ratio M-H, Fixed, 95% CI	Risk of Bias A	B	C	D	E	F
**10.14.1 Tirzepatide**												
Garvey 2023	0	623	1	315	9.3%	0.17 [0.01, 4.14]	+	+	+	?	?	?
Jastreboff 2022	1	1896	0	643	3.5%	1.02 [0.04, 25.03]	+	+	+	+	?	?
Loomba 2024	0	95	3	48	21.7%	0.07 [0.00, 1.35]	+	+	+	?	?	?
Malhotra 2024	2	234	3	235	13.9%	0.67 [0.11, 4.03]	+	+	+	?	?	?
**Subtotal (95% CI)**		**2848**		**1241**	**48.4%**	**0.33 [0.10, 1.04]**						
Total events	3		7									
Heterogeneity: Chi² = 2.31, df = 3 (P = 0.51); I² = 0%												
Test for overall effect: Z = 1.89 (P = 0.06)												
**10.14.2 GLP1-RA**												
Lincoff 2023	2	8803	6	8801	28.2%	0.33 [0.07, 1.65]	+	+	+	?	?	?
Rubino 2021	1	253	0	85	3.5%	1.02 [0.04, 25.17]	+	?	?	+	?	?
Wadden 2013	0	212	1	206	7.1%	0.32 [0.01, 7.96]	+	+	+	+	?	?
**Subtotal (95% CI)**		**9268**		**9092**	**38.8%**	**0.39 [0.11, 1.39]**						
Total events	3		7									
Heterogeneity: Chi² = 0.39, df = 2 (P = 0.82); I² = 0%												
Test for overall effect: Z = 1.45 (P = 0.15)												
**10.14.3 Surgery-Endoscopy**												
Abu Dayyeh 2022	0	77	2	110	9.6%	0.28 [0.01, 5.91]	+	?	?	?	?	?
**Subtotal (95% CI)**		**77**		**110**	**9.6%**	**0.28 [0.01, 5.91]**						
Total events	0		2									
Heterogeneity: Not applicable												
Test for overall effect: Z = 0.82 (P = 0.41)												
**10.14.4 Bupropione/Naltrexone**												
Apovian 2013	3	992	0	492	3.1%	3.48 [0.18, 67.58]	+	+	?	+	+	?
**Subtotal (95% CI)**		**992**		**492**	**3.1%**	**3.48 [0.18, 67.58]**						
Total events	3		0									
Heterogeneity: Not applicable												
Test for overall effect: Z = 0.83 (P = 0.41)												
**Total (95% CI)**		**13185**		**10935**	**100.0%**	**0.45 [0.21, 0.94]**						
Total events	9		16									
Heterogeneity: Chi² = 4.68, df = 8 (P = 0.79); I² = 0%												
Test for overall effect: Z = 2.13 (P = 0.03)												
Test for subgroup differences: Chi² = 2.21, df = 3 (P = 0.53), I² = 0%												

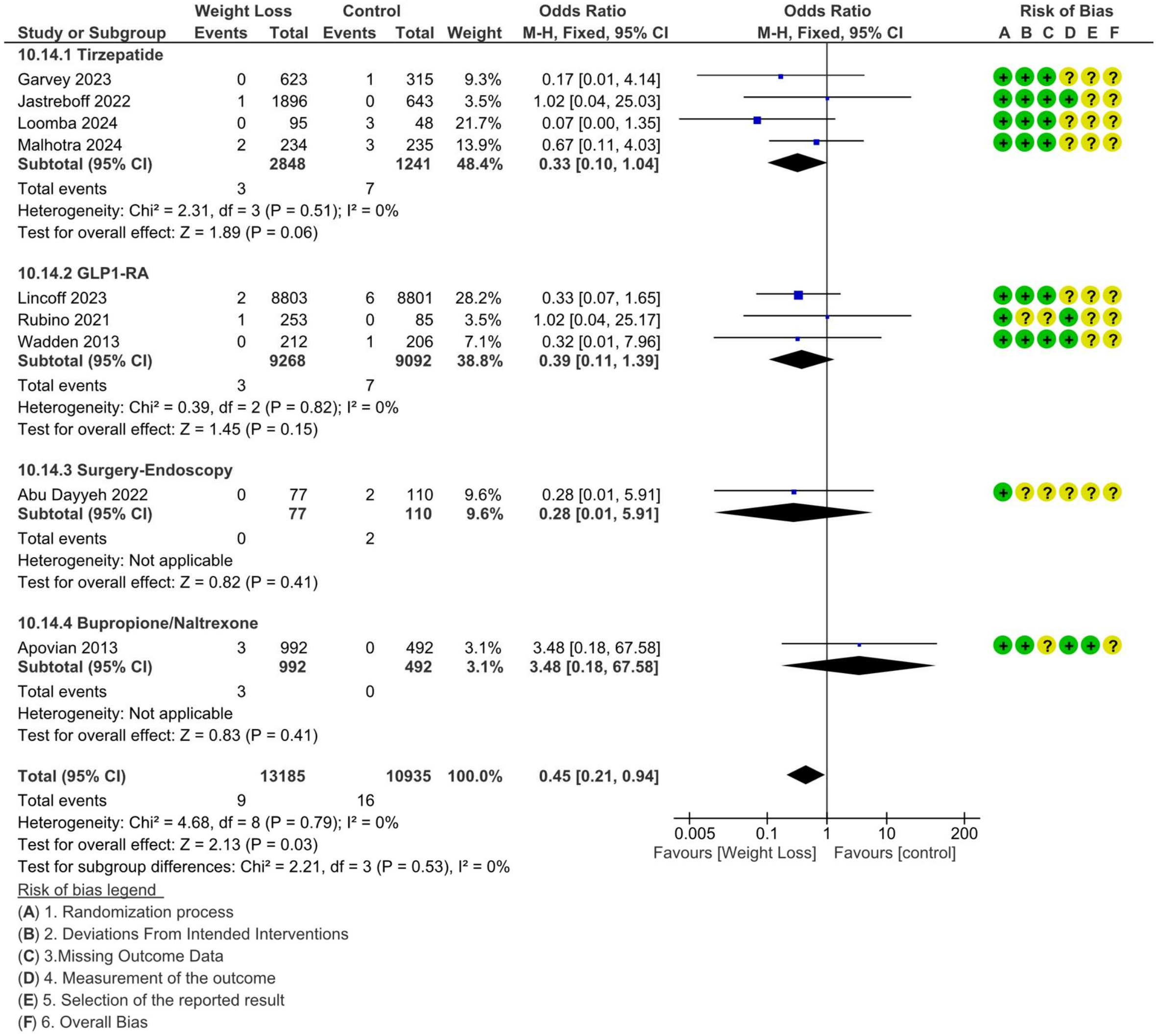

Risk of bias legend
(**A**) 1. Randomization process
(**B**) 2. Deviations From Intended Interventions
(**C**) 3.Missing Outcome Data
(**D**) 4. Measurement of the outcome
(**E**) 5. Selection of the reported result
(**F**) 6. Overall Bias

Figure S14: Incidence of Major Depression, subgroup analysis by interventions

Study or Subgroup	Buproprione/Naltrexone Mean	SD	Total	Control Mean	SD	Total	Weight	Mean Difference IV, Fixed, 95% CI	Risk of Bias A	B	C	D	E	F
Apovian 2013	6.7	5.9	992	6.4	5.3	492	38.1%	0.30 [-0.30, 0.90]	+	+	?	+	+	?
Greenway 2010	6.4	5.5	1142	5.5	5	569	49.8%	0.90 [0.38, 1.42]	+	+	+	+	+	+
Hollander 2013	8.5	5.7	333	6.3	5.7	169	12.1%	2.20 [1.14, 3.26]	+	?	+	+	+	?
**Total (95% CI)**			**2467**			**1230**	**100.0%**	**0.83 [0.46, 1.20]**						
Heterogeneity: Chi² = 9.59, df = 2 (P = 0.008); I² = 79%														
Test for overall effect: Z = 4.43 (P < 0.00001)														

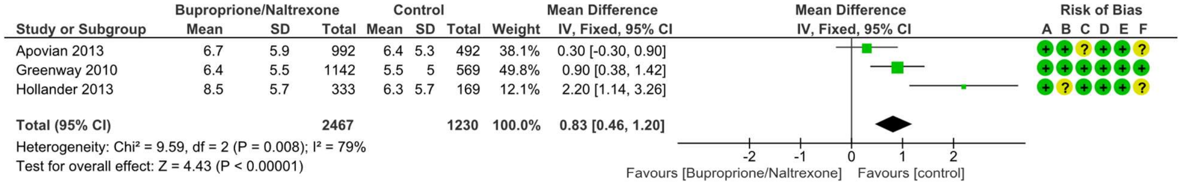

Risk of bias legend
(**A**) 1. Randomization process
(**B**) 2. Deviations From Intended Interventions
(**C**) 3.Missing Outcome Data
(**D**) 4. Measurement of the outcome
(**E**) 5. Selection of the reported result
(**F**) 6. Overall Bias

Figure S15: Improvement in Inventory Depression Scale (IDS) scores

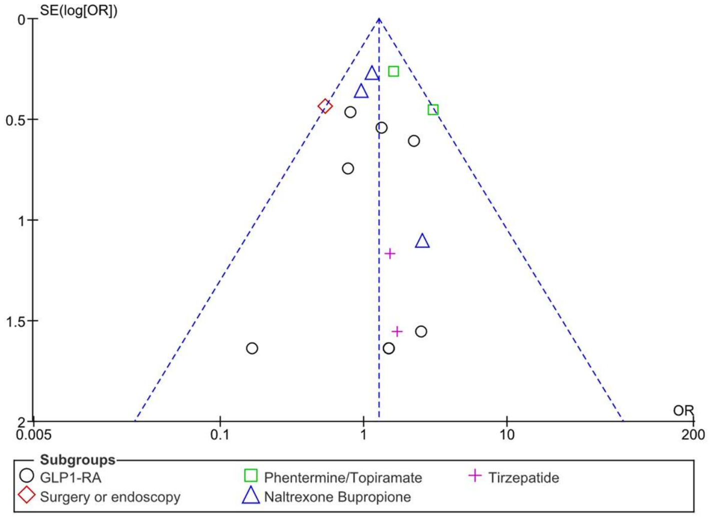

Figure S16: Funnel plot (Risk for Anxiety)

Study or Subgroup	Weight Loss Events	Weight Loss Total	Control Events	Control Total	Weight	Odds Ratio M-H, Fixed, 95% CI	A	B	C	D	E	F
**10.15.1 GLP1-RA**												
Bliddal 2024	0	271	0	136		Not estimable	+	+	+	?	?	?
Davies 2015	1	423	0	212	0.7%	1.51 [0.06, 37.20]	+	+	+	+	?	?
Garvey 2022	9	152	11	152	11.7%	0.81 [0.32, 2.01]	+	+	+	?	?	+
Leroux 2017	0	1505	1	749	2.3%	0.17 [0.01, 4.07]	+	+	+	+	+	+
Lincoff 2023	8	8803	6	8801	6.8%	1.33 [0.46, 3.84]	+	+	+	?	?	?
O' Neill 2018	3	103	5	136	4.7%	0.79 [0.18, 3.37]	+	+	+	+	?	+
Wadden 2013	9	212	4	206	4.4%	2.24 [0.68, 7.39]	+	+	+	+	?	?
Wadden 2021	1	407	0	204	0.7%	1.51 [0.06, 37.21]	+	+	+	+	+	+
Wilding 2021	2	1306	0	655	0.8%	2.51 [0.12, 52.41]	+	+	+	+	+	+
**Subtotal (95% CI)**		**13182**		**11251**	**32.1%**	**1.14 [0.69, 1.89]**						
Total events	33		27									
Heterogeneity: Chi² = 3.83, df = 7 (P = 0.80); I² = 0%												
Test for overall effect: Z = 0.50 (P = 0.61)												
**10.15.2 Surgery**												
Cohen 2020	13	51	19	49	16.3%	0.54 [0.23, 1.27]	+	?	?	+	+	?
**Subtotal (95% CI)**		**51**		**49**	**16.3%**	**0.54 [0.23, 1.27]**						
Total events	13		19									
Heterogeneity: Not applicable												
Test for overall effect: Z = 1.42 (P = 0.16)												
**10.15.4 Naltr Brup**												
Apovian 2013	48	992	21	492	30.2%	1.14 [0.67, 1.93]	+	+	?	+	+	?
Greenway 2010	23	1142	12	569	17.7%	0.95 [0.47, 1.93]	+	+	+	+	+	+
Hollander 2013	5	333	1	169	1.5%	2.56 [0.30, 22.10]	+	?	+	+	+	?
**Subtotal (95% CI)**		**2467**		**1230**	**49.3%**	**1.12 [0.74, 1.68]**						
Total events	76		34									
Heterogeneity: Chi² = 0.77, df = 2 (P = 0.68); I² = 0%												
Test for overall effect: Z = 0.52 (P = 0.60)												
**10.15.6 Tirzepatide**												
Jastreboff 2022	2	1896	0	643	0.8%	1.70 [0.08, 35.42]	+	+	+	+	?	?
Loomba 2024	3	95	1	48	1.5%	1.53 [0.16, 15.14]	+	+	+	?	?	?
**Subtotal (95% CI)**		**1991**		**691**	**2.3%**	**1.59 [0.26, 9.91]**						
Total events	5		1									
Heterogeneity: Chi² = 0.00, df = 1 (P = 0.96); I² = 0%												
Test for overall effect: Z = 0.50 (P = 0.62)												
**Total (95% CI)**		**17691**		**13221**	**100.0%**	**1.04 [0.78, 1.39]**						
Total events	127		81									
Heterogeneity: Chi² = 7.26, df = 13 (P = 0.89); I² = 0%												
Test for overall effect: Z = 0.26 (P = 0.79)												
Test for subgroup differences: Chi² = 2.71, df = 3 (P = 0.44), I² = 0%												

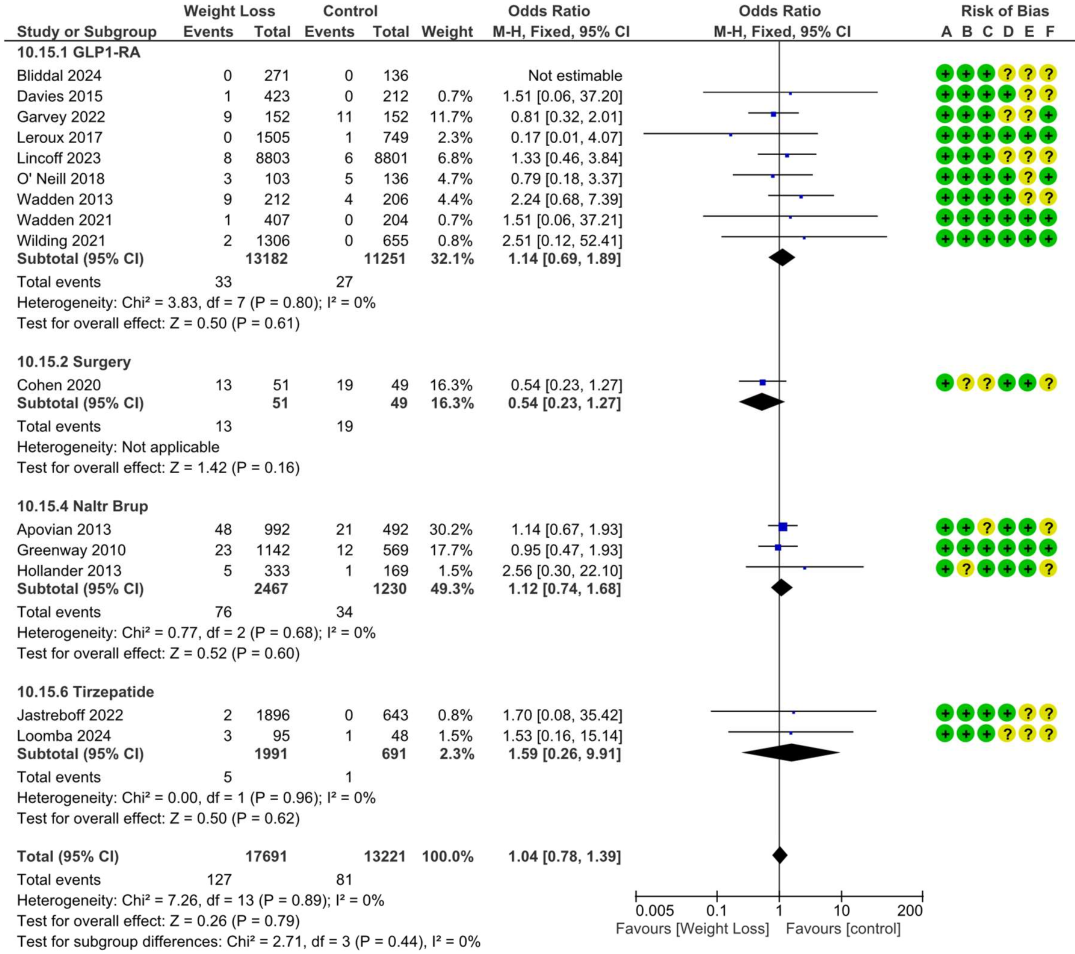

Risk of bias legend
(**A**) 1. Randomization process
(**B**) 2. Deviations From Intended Interventions
(**C**) 3.Missing Outcome Data
(**D**) 4. Measurement of the outcome
(**E**) 5. Selection of the reported result
(**F**) 6. Overall Bias

Fig. S17: Difference in risk in Anxiety between weight loss and controls, subgroup analysis by intervention (EMA -approved drugs).

Study or Subgroup	GLP-1RA Events	GLP-1RA Total	Control Events	Control Total	Weight	Odds Ratio M-H, Fixed, 95% CI	Risk of Bias A B C D E F
**10.15.1 GLP1-RA**							
Wilding 2021	2	1306	0	655	0.6%	2.51 [0.12, 52.41]	+ + + + + +
Wadden 2021	1	407	0	204	0.6%	1.51 [0.06, 37.21]	+ + + + + +
Wadden 2013	9	212	4	206	3.2%	2.24 [0.68, 7.39]	+ + + + ? ?
O' Neill 2018	3	103	5	136	3.5%	0.79 [0.18, 3.37]	+ + + + ? +
Lincoff 2023	8	8803	6	8801	5.0%	1.33 [0.46, 3.84]	+ + + ? ? ?
Leroux 2017	0	1505	1	749	1.7%	0.17 [0.01, 4.07]	+ + + + + +
Garvey 2022	9	152	11	152	8.6%	0.81 [0.32, 2.01]	+ + + ? ? +
Davies 2015	1	423	0	212	0.6%	1.51 [0.06, 37.20]	+ + + + ? ?
Bliddal 2024	0	271	0	136		Not estimable	+ + + ? ? ?
**Subtotal (95% CI)**		**13182**		**11251**	**23.7%**	**1.14 [0.69, 1.89]**	
Total events	33		27				
Heterogeneity: Chi² = 3.83, df = 7 (P = 0.80); I² = 0%							
Test for overall effect: Z = 0.50 (P = 0.61)							
**10.15.2 Surgery or endoscopy**							
Cohen 2020	13	51	19	49	12.0%	0.54 [0.23, 1.27]	+ ? ? + + ?
**Subtotal (95% CI)**		**51**		**49**	**12.0%**	**0.54 [0.23, 1.27]**	
Total events	13		19				
Heterogeneity: Not applicable							
Test for overall effect: Z = 1.42 (P = 0.16)							
**10.15.3 Phentermine/Topiramate**							
Gadde 2011	50	1492	21	993	20.3%	1.60 [0.96, 2.69]	+ + + + + +
Allison 2012	26	751	6	513	5.7%	3.03 [1.24, 7.42]	+ + ? + + ?
**Subtotal (95% CI)**		**2243**		**1506**	**26.1%**	**1.92 [1.23, 2.99]**	
Total events	76		27				
Heterogeneity: Chi² = 1.46, df = 1 (P = 0.23); I² = 32%							
Test for overall effect: Z = 2.88 (P = 0.004)							
**10.15.4 Naltrexone Bupropione**							
Hollander 2013	5	333	1	169	1.1%	2.56 [0.30, 22.10]	+ ? + + + ?
Greenway 2010	23	1142	12	569	13.1%	0.95 [0.47, 1.93]	+ + + + + +
Apovian 2013	48	992	21	492	22.3%	1.14 [0.67, 1.93]	+ + ? + + ?
**Subtotal (95% CI)**		**2467**		**1230**	**36.5%**	**1.12 [0.74, 1.68]**	
Total events	76		34				
Heterogeneity: Chi² = 0.77, df = 2 (P = 0.68); I² = 0%							
Test for overall effect: Z = 0.52 (P = 0.60)							
**10.15.6 Tirzepatide**							
Loomba 2024	3	95	1	48	1.1%	1.53 [0.16, 15.14]	+ + + ? ? ?
Jastreboff 2022	2	1896	0	643	0.6%	1.70 [0.08, 35.42]	+ + + + ? ?
**Subtotal (95% CI)**		**1991**		**691**	**1.7%**	**1.59 [0.26, 9.91]**	
Total events	5		1				
Heterogeneity: Chi² = 0.00, df = 1 (P = 0.96); I² = 0%							
Test for overall effect: Z = 0.50 (P = 0.62)							
**Total (95% CI)**		**19934**		**14727**	**100.0%**	**1.27 [1.00, 1.62]**	
Total events	203		108				
Heterogeneity: Chi² = 13.56, df = 15 (P = 0.56); I² = 0%							
Test for overall effect: Z = 1.93 (P = 0.05)							
Test for subgroup differences: Chi² = 7.79, df = 4 (P = 0.10), I² = 48.7%							

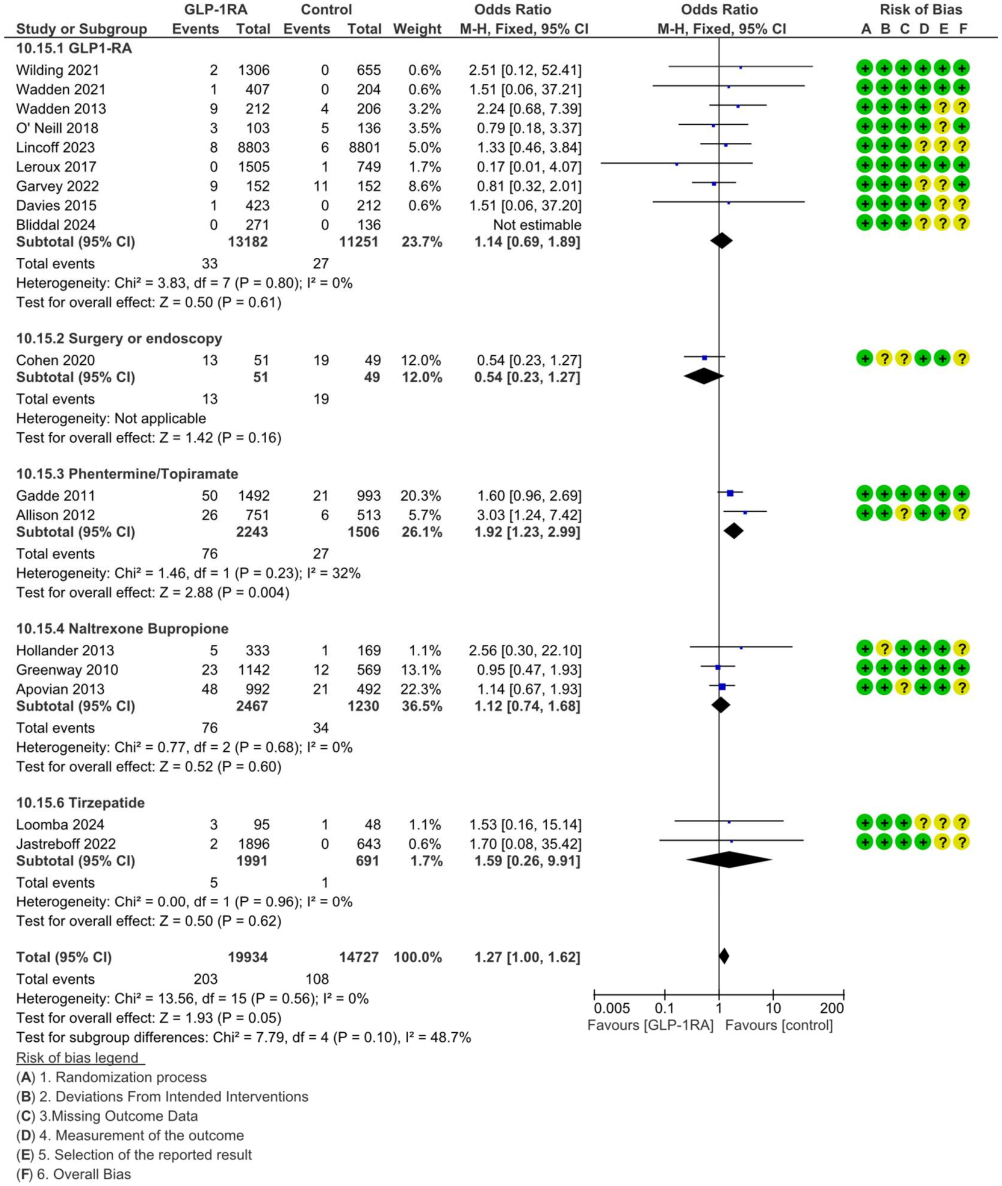

Risk of bias legend

(**A**) 1. Randomization process
(**B**) 2. Deviations From Intended Interventions
(**C**) 3.Missing Outcome Data
(**D**) 4. Measurement of the outcome
(**E**) 5. Selection of the reported result
(**F**) 6. Overall Bias

Fig. S18: Difference in risk in Anxiety between weight loss and controls, subgroup analysis by intervention, (FDA & EMA -approved drugs)

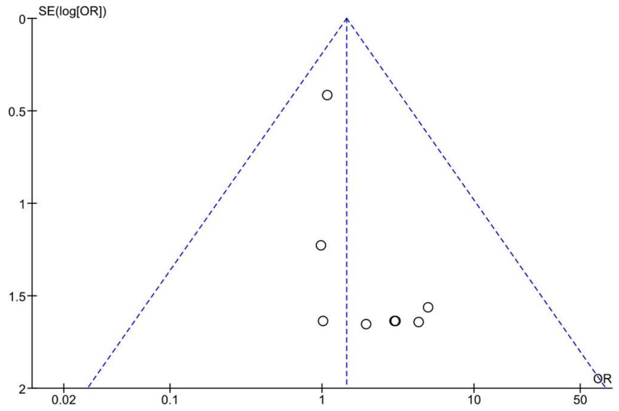

Figure S19: Funnel plot (Incidence of suicidality)

Study or Subgroup	Weight Loss Events	Weight Loss Total	Control Events	Control Total	Weight	Odds Ratio M-H, Fixed, 95% CI	Risk of Bias A B C D E F
**10.7.1 Tirzepatide**							
Garvey 2023	0	623	1	315	11.1%	0.17 [0.01, 4.14]	+ + + ? ? ?
Jastreboff 2022	3	1896	0	643	4.1%	2.38 [0.12, 46.12]	+ + + + ? ?
Malhotra 2024	2	234	0	235	2.7%	5.06 [0.24, 106.06]	+ + + ? ? ?
**Subtotal (95% CI)**		**2753**		**1193**	**18.0%**	**1.43 [0.33, 6.11]**	
Total events	5		1				
Heterogeneity: Chi² = 2.49, df = 2 (P = 0.29); I² = 20%							
Test for overall effect: Z = 0.48 (P = 0.63)							
**10.7.2 GLP1-RA**							
Garvey 2022	0	152	1	152	8.3%	0.33 [0.01, 8.19]	+ + + ? ? +
Leroux 2017	2	1505	1	749	7.4%	1.00 [0.09, 10.99]	+ + + + + +
Lincoff 2023	7	8803	8	8801	44.5%	0.87 [0.32, 2.41]	+ + + ? ? ?
Wilding 2021	1	1306	1	655	7.4%	0.50 [0.03, 8.02]	+ + + + + +
**Subtotal (95% CI)**		**11766**		**10357**	**67.6%**	**0.78 [0.33, 1.82]**	
Total events	10		11				
Heterogeneity: Chi² = 0.46, df = 3 (P = 0.93); I² = 0%							
Test for overall effect: Z = 0.58 (P = 0.56)							
**10.7.3 Naltrexone-Bupropione**							
Apovian 2013	0	992	1	492	11.1%	0.17 [0.01, 4.06]	+ + ? + + ?
**Subtotal (95% CI)**		**992**		**492**	**11.1%**	**0.17 [0.01, 4.06]**	
Total events	0		1				
Heterogeneity: Not applicable							
Test for overall effect: Z = 1.10 (P = 0.27)							
**10.7.4 Surgery-Endoscopy**							
Caiazzo 2020	1	49	0	31	3.3%	1.95 [0.08, 49.35]	? ? ? ? ? ?
**Subtotal (95% CI)**		**49**		**31**	**3.3%**	**1.95 [0.08, 49.35]**	
Total events	1		0				
Heterogeneity: Not applicable							
Test for overall effect: Z = 0.40 (P = 0.69)							
**Total (95% CI)**		**15560**		**12073**	**100.0%**	**0.87 [0.44, 1.70]**	
Total events	16		13				
Heterogeneity: Chi² = 4.53, df = 8 (P = 0.81); I² = 0%							
Test for overall effect: Z = 0.42 (P = 0.68)							
Test for subgroup differences: Chi² = 1.78, df = 3 (P = 0.62), I² = 0%							

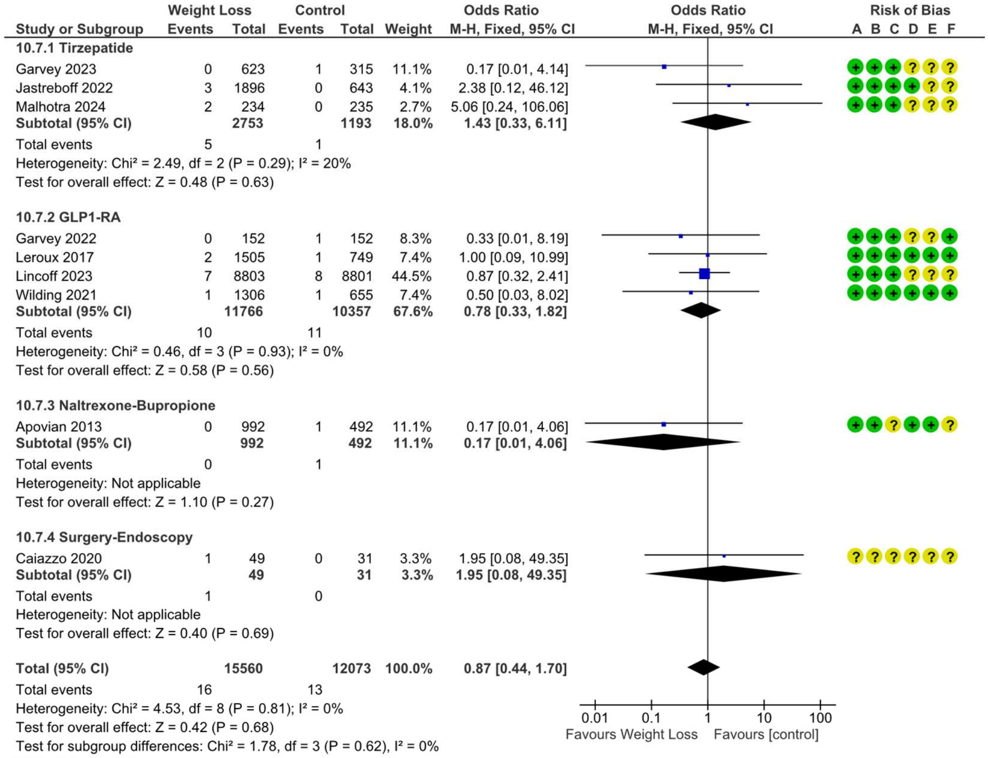

Risk of bias legend
(**A**) 1. Randomization process
(**B**) 2. Deviations From Intended Interventions
(**C**) 3.Missing Outcome Data
(**D**) 4. Measurement of the outcome
(**E**) 5. Selection of the reported result
(**F**) 6. Overall Bias

Fig. S20: Difference in the incidence of Suicidal Ideation between weight loss and controls, subgroup analysis by intervention, (FDA & EMA - approved interventions)

Study or Subgroup	Weight Loss Events	Weight Loss Total	Control Events	Control Total	Weight	Odds Ratio M-H, Fixed, 95% CI	Risk of Bias A B C D E F
Apovian 2013	0	992	1	492	50.1%	0.17 [0.01, 4.06]	+ + ? + + ?
Lincoff 2023	5	8803	2	8801	49.9%	2.50 [0.48, 12.89]	+ + + ? ? ?
**Total (95% CI)**		**9795**		**9293**	**100.0%**	**1.33 [0.36, 4.91]**	
Total events	5		3				
Heterogeneity: Chi² = 2.20, df = 1 (P = 0.14); I² = 55%							
Test for overall effect: Z = 0.43 (P = 0.67)							

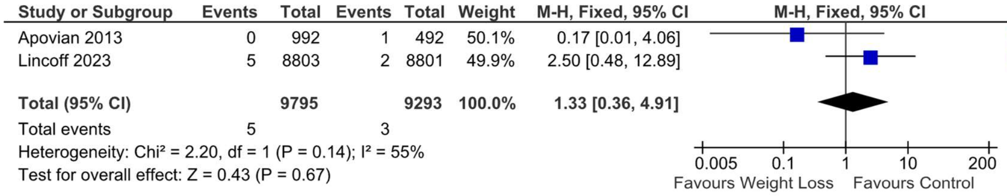

Risk of bias legend
(**A**) 1. Randomization process
(**B**) 2. Deviations From Intended Interventions
(**C**) 3.Missing Outcome Data
(**D**) 4. Measurement of the outcome
(**E**) 5. Selection of the reported result
(**F**) 6. Overall Bias

Fig. S21: Difference in the incidence of Suicide between weight loss and controls

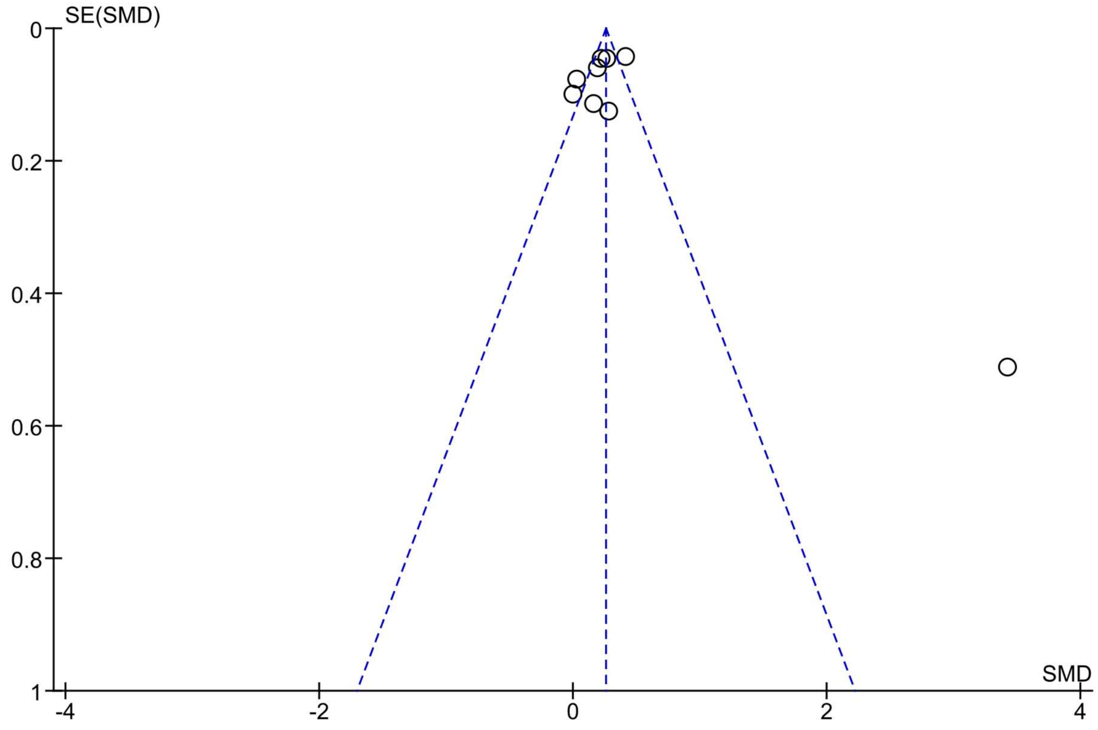

Figure S20: Funnel plot sf-36 Physical function

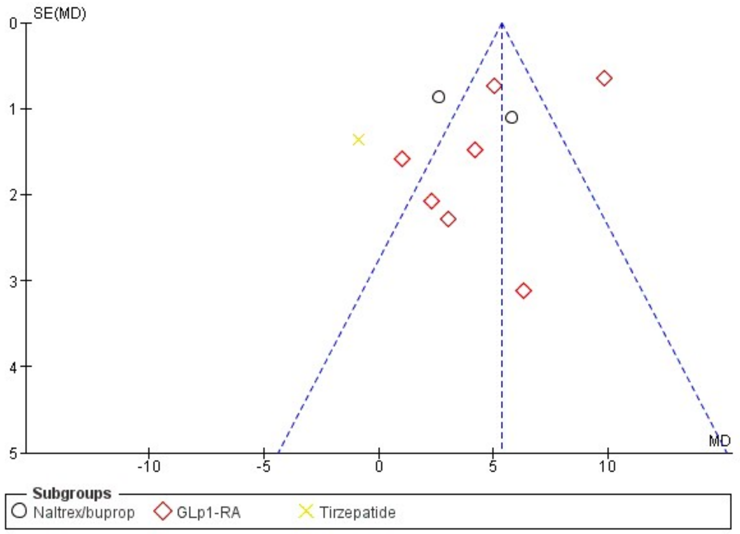

Figure S21: Funnel plot IWQOL LITE

Certainty assessment							№ of patients		Effect		Certainty
№ of studies	Study design	Risk of bias	Inconsistency	Indirectness	Imprecision	Other considerations	Weight loss	control	Relative (95% CI)	Absolute (95% CI)	
**Depression**											
16	randomised trials	Serious	not serious	not serious	not serious	none	99/16405 (0.6%)	88/12619 (0.7%)	**OR 0.72** (0.54 to 0.97)	**2 fewer per 1.000** (from 3 fewer to 0 fewer)	⨁⨁⨁◯ Moderate
**Psichiatric Serious Adverse Events**											
16	randomised trials	not serious	not serious	not serious	serious[a]	none	83/15375 (0.5%)	66/12595 (0.5%)	**OR 1.07** (0.78 to 1.47)	**0 fewer per 1.000** (from 1 fewer to 2 more)	⨁⨁⨁◯ Moderate[a]
**Suicidal ideation**											
9	randomised trials	not serious	not serious	not serious	serious[a]	none	16/15560 (0.1%)	13/12073 (0.1%)	**OR 0.87** (0.44 to 1.70)	**0 fewer per 1.000** (from 1 fewer to 1 more)	⨁⨁⨁◯ Moderate[a]
**Major Depression**											
9	randomised trials	not serious	not serious	not serious	serious[a]	None	9/13185 (0.1%)	16/10935 (0.1%)	**OR 0.45** (0.21 to 0.94)	**1 fewer per 1.000** (from 1 fewer to 0 fewer)	⨁⨁⨁◯ Moderate[a]
**Anxiety**											
15	randomised trials	serious	not serious	not serious	not serious	None	127/17691 (0.7%)	81/13221 (0.6%)	**OR 1.04** (0.78 to 1.39)	**0 fewer per 1.000** (from 1 fewer to 2 more)	⨁⨁⨁◯ Moderate
**Short Form-36 Mental**											
4	randomised trials	not serious	serious[b]	not serious	not serious	None	1982	1016	-	SMD **0.45 higher** (0.37 higher to 0.52 higher)	⨁⨁⨁◯ Moderate[b]
**Impact Weight Quality Of Life Lite Physical functIon**											
10	randomised trials	not serious	serious[b]	not serious[b]	not serious[a]	publication bias suspected[c]	7097	9711	-	MD **3.96 higher** (1.6 higher to 6.32 higher)	⨁⨁◯◯ Low[a,b,c]

Table 3S: GRADE Assessment CI: confidence interval; MD: mean difference; OR: odds ratio; SMD: standardised mean difference. Explanations: a. Small number of events b. Significant Heterogeneity c. Funnel plot does not rule out publication bias
